# Hyperglycemia and O-GlcNAc transferase activity drive a cancer stem cell pathway in triple-negative breast cancer

**DOI:** 10.1186/s12935-023-02942-6

**Published:** 2023-05-25

**Authors:** Saheed A. Ayodeji, Bin Bao, Emily A. Teslow, Lisa A. Polin, Greg Dyson, Aliccia Bollig-Fischer, Charlie Fehl

**Affiliations:** 1 Department of Chemistry, Wayne State University, 5101 Cass Avenue, Detroit, MI USA; 2 Department of Oncology, Barbara Ann Karmanos Cancer Institute, Wayne State University School of Medicine, Detroit, MI 48201 USA

**Keywords:** O-GlcNAc transferase, Hyperglycemia, TNBC, Tumorigenesis, Metabolic disease, Glycobiology, Chemical biology, Obesity, TET1, Epigenetics

## Abstract

**Background:**

Enhanced glucose metabolism is a feature of most tumors, but downstream functional effects of aberrant glucose flux are difficult to mechanistically determine. Metabolic diseases including obesity and diabetes have a hyperglycemia component and are correlated with elevated pre-menopausal cancer risk for triple-negative breast cancer (TNBC). However, determining pathways for hyperglycemic disease-coupled cancer risk remains a major unmet need. One aspect of cellular sugar utilization is the addition of the glucose-derived protein modification O-GlcNAc (O-linked N-acetylglucosamine) via the single human enzyme that catalyzes this process, O-GlcNAc transferase (OGT). The data in this report implicate roles of OGT and O-GlcNAc within a pathway leading to cancer stem-like cell (CSC) expansion. CSCs are the minor fraction of tumor cells recognized as a source of tumors as well as fueling metastatic recurrence. The objective of this study was to identify a novel pathway for glucose-driven expansion of CSC as a potential molecular link between hyperglycemic conditions and CSC tumor risk factors.

**Methods:**

We used chemical biology tools to track how a metabolite of glucose, GlcNAc, became linked to the transcriptional regulatory protein tet-methylcytosine dioxygenase 1 (TET1) as an O-GlcNAc post-translational modification in three TNBC cell lines. Using biochemical approaches, genetic models, diet-induced obese animals, and chemical biology labeling, we evaluated the impact of hyperglycemia on CSC pathways driven by OGT in TNBC model systems.

**Results:**

We showed that OGT levels were higher in TNBC cell lines compared to non-tumor breast cells, matching patient data. Our data identified that hyperglycemia drove O-GlcNAcylation of the protein TET1 via OGT-catalyzed activity. Suppression of pathway proteins by inhibition, RNA silencing, and overexpression confirmed a mechanism for glucose-driven CSC expansion via TET1-O-GlcNAc. Furthermore, activation of the pathway led to higher levels of OGT production via feed-forward regulation in hyperglycemic conditions. We showed that diet-induced obesity led to elevated tumor OGT expression and O-GlcNAc levels in mice compared to lean littermates, suggesting relevance of this pathway in an animal model of the hyperglycemic TNBC microenvironment.

**Conclusions:**

Taken together, our data revealed a mechanism whereby hyperglycemic conditions activated a CSC pathway in TNBC models. This pathway can be potentially targeted to reduce hyperglycemia-driven breast cancer risk, for instance in metabolic diseases. Because pre-menopausal TNBC risk and mortality are correlated with metabolic diseases, our results could lead to new directions including OGT inhibition for mitigating hyperglycemia as a risk factor for TNBC tumorigenesis and progression.

**Supplementary Information:**

The online version contains supplementary material available at 10.1186/s12935-023-02942-6.

## Background

Glucose uptake is aberrantly high in most cancers because of altered metabolism and growth kinetics [1, 2]. Tumor cells are well established to divert glucose into aerobic glycolysis as a way to synthesize the requisite biosynthetic intermediates for rapid cell division, known as the Warburg Effect [2–4]. In the systemic context, a key source of sustenance for tumors is elevated blood glucose, which leads to a hyperglycemic microenvironment able to promote angiogenesis, chemoresistance, and growth [1]. Conversely, caloric restriction minimizes the hyperglycemic microenvironment and can reduce breast cancer growth as well as metastasis [5]. These glucose-centered studies reveal a link between blood sugar and tumor growth. However, discrete molecular targets for hyperglycemic cancer risk remain difficult to mechanistically determine because of the many cellular pathways affected by increased glucose flux [6]. As the prevalence of metabolic disease rises worldwide, so too is the cancer burden increasing [7]. The anti-hyperglycemic diabetes drug metformin has completed several clinical trials, but hyperglycemia-associated treatment resistance continues to pose a challenge even in metformin-enhanced chemotherapy [1, 8]. Determining effective, therapeutically targetable mechanisms of hyperglycemia-driven cancer pathways remains a critical unmet medical challenge [9].

**Fig. 1:**
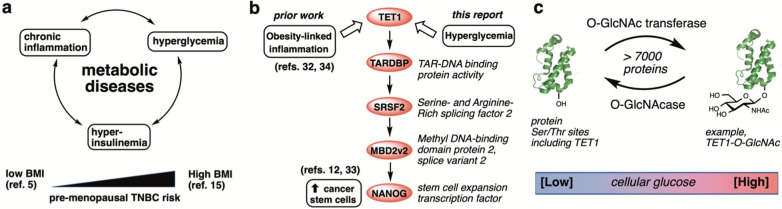
Pre-menopausal TNBC risk relates to metabolic disease aspects including hyperglycemia. **a** Conserved facets of metabolic diseases and positive association of pre-menopausal TNBC risk with body-mass index (BMI). **b** The pathway under study for hyperglycemia driven TNBC tumorigenesis, beginning with the epigenetic regulator tet methylcytosine dioxygenase 1 (TET1). **c** O-GlcNAc glycosylation is a widespread glucose sensing protein post-translational modification regulated by just two enzymes, the O-GlcNAc transferase (OGT) and the O-GlcNAcase (OGA)

Metabolic diseases including obesity and type 2 diabetes have been specifically linked with higher risk for triple-negative breast cancer (TNBC) in pre-menopausal women by both targeted [10–12] and metanalytical [13–17] studies. A TNBC diagnosis means that the tumor cancer cells lack expression of two key hormone receptors and a growth factor receptor: the estrogen receptor ERα, the progesterone receptor, and the human epidermal growth factor receptor (HER2), a tyrosine kinase [18]. Obesity and type 2 diabetes share several common features including hyperglycemia, insulin resistance, and chronic low-grade inflammation (Fig. 1a) [19–22]. In pre-menopausal women, these three pathological features of metabolic diseases downregulate the expression of ERα [23, 24], lowering risk of hormone-receptor positive BC but paradoxically increasing the risk for TNBC [17, 22, 25]. Estimates for the increased risk of early onset TNBC in diabetic patients are roughly 28% [11, 13, 26]. The increased risk in medically obese or overweight patients is also significant, estimated by two distinct studies to be between 43 and 80% higher in obese or overweight pre-menopausal women [15, 27].

TNBC is concerning because it lacks antihormone treatment options normally available to breast cancer patients [18]. TNBC tumors are, however, characterized to have a greater fraction of cancer stem-like cells (CSCs) than hormone-positive BC tumors [28, 29]. CSCs are recognized as the subpopulation of tumor cells that give rise to malignant tumorigenesis [30, 31]. Along this line of inquiry, we published a connection between obesity-induced inflammation and TNBC tumorigenesis through a novel pathway to CSC expansion [12, 32, 33]. Briefly, the inflammatory signaling molecule interleukin 6 (IL-6) triggers production of reactive oxygen species and activates the oxidative enzyme tet methylcytosine dioxygenase 1 (TET1). TET1 initiates a cascade to CSC induction via the proteins in Fig. 1b, culminating in the stem cell inducing factor NANOG. This pathway is active in diet-induced obese mouse models of TNBC, showing that obesity-associated inflammatory signals activate the TET1-NANOG system in vivo [12, 32]. As defined by body mass index (BMI), TET1 and downstream pathway proteins TARDBP (TAR DNA binding protein), SRSF2 (serine and arginine-rich splicing factor 2), and MBD2_v2 (methyl DNA-binding protein domain protein, oncogenic splice variant 2) RNA levels are also significantly overexpressed in obese (BMI > 30 kg/m^2^) vs. non-obese (BMI < 30) human patient samples by 3- to 11-fold [12, 32]. RNA levels for the critical CSC factor NANOG are elevated by 8-fold in obese (BMI > 30) patients compared to non-obese (BMI < 30) patients [32]. Supporting this pathway, antioxidant treatment overcomes TNBC chemoresistance specifically via downregulating the CSC subpopulation of TNBC tumors in mice [34]. However, a remaining gap in our pathway is a role for hyperglycemia, another intrinsic facet of obesity. We hypothesized that CSC pathways connect metabolic disease with pre-menopausal TNBC tumorigenic risk because other BC subtypes are not reliant on high CSC populations.

Intriguingly, TET1, which mediates the inflammation/reactive oxygen effects on TNBC CSC expansion, is also well-characterized as a glucose sensor through its interaction with the sugar signaling enzyme O-GlcNAc transferase (OGT) [35–37]. OGT modifies proteins including TET1 at serine and threonine sites by O-linked N-acetylglucosamine (O-GlcNAc), a glucose-derived post-translational modification found on >7000 proteins (Fig. 1c) [38, 39]. Tumors display aberrantly high O-GlcNAcylation [40], a result of Warburg Effect glucose uptake and the hyperglycemic tumor microenvironment because ca. 2–5% of cellular glucose is converted by the hexosamine biosynthetic pathway to the OGT sugar substrate UDP-GlcNAc (uridine diphosphate-N-acetylglucosamine) [41–43]. Elevated overall O-GlcNAc levels therefore enable O-GlcNAcylated protein functions to serve biochemical nutrient-sensing roles in cells [41]. There is a growing association between O-GlcNAc protein modifications and enhanced cancer progression [40, 44, 45], but our mechanistic understanding of O-GlcNAc-driven tumors remains far from solved because over 7000 proteins are known to be O-GlcNAc modified and OGT is the single enzyme responsible for all protein O-GlcNAc modifications [46, 47]. This extreme reliance on a single protein for all O-GlcNAc protein modifications makes OGT’s effects on particular pathways difficult to determine [48]. However, key effects of OGT have been observed in breast cancers including activation of the oncogenic transcription factors FoxM1 [49] and a strong overexpression in breast cancer stem-like cells [50]. Also, between breast cancer subtypes, OGT inhibition is most effective in TNBC [51]. We therefore expanded our hypothesis for metabolic disease-driven TNBC tumorigenesis to include hyperglycemic activation of our published TET1 CSC pathway via the physical and biochemical connection of TET1 with OGT [37].

The focus of this study was to determine defining features of breast cancer subtypes that connect hyperglycemia with TNBC tumorigenesis (Fig. 1b). Chemical biology labeling data revealed that OGT directly modified TET1 with O-GlcNAc in TNBC cell lines. Glucose-driven TET1-O-GlcNAcylation led to higher expression levels of TET1 target genes, including those in the pathway reported by us leading to CSCs [12, 32]. Furthermore, our data revealed that hyperglycemic cell conditions enhanced OGT activity and expression in TNBC cell lines as well as TNBC tumors grown in diet-induced obese mouse models. Using inhibitors, RNA silencing, and overexpression engineering, we determined a potential hyperglycemia-promoted mechanism for upregulated OGT levels in TNBC that involves TET1. Taken together, our data suggest that OGT-catalyzed O-GlcNAcylation may be a targetable link between hyperglycemia-associated diseases and TNBC risk through a CSC pathway that affects TNBC tumorigenesis. This study is the first to report evidence that high glucose elevates O-GlcNAcylation of TET1 via increased OGT activity to activate a known TET1-driven CSC expansion pathway.

## Methods

### Cell Culture and reagents

MDA-MB-231, MDA-MB-468, HCC70, BT474, and T47D cells were originally purchased from ATCC. MCF 10 A was obtained from Karmanos Cancer Institute (former Michigan Cancer Foundation). All cells were verified to be free from mycoplasma using the MycoAlert^™^ Mycoplasma Detection kit (LT0-118, Lonza group Ltd.) and MycoAlert^™^ control set (LT07-518, Lonza group Ltd.). The cells were all cultured in humidified sterile incubator conditioned at 5% CO_2_ at 37 ^o^C. BT474 cells were grown in Hybricare medium (ATCC 46-X) supplemented with 10% FBs and 1% Pen/strep. MDA-MB-231 and MDA-MB-468 cells were grown in high glucose (4.5 g/L) or low glucose (1.0 g/L) DMEM media (ThermoFisher) supplemented with 10% Fetal Bovine Serum (FBS) and 1% Penicillin/Streptavidin (Pen/Strep). HCC70 and T47D cells were cultured in high glucose (4.5 g/L) or low glucose (1.0 g/L) RPMI-1640 media (ThermoFisher) supplemented with 10% FBS and 1% Pen/Strep. MCF 10 A cells were cultured in a 1:1 DMEM: F-12 high glucose medium supplemented with 5% horse serum, 20 ng/mL EGF, 0.5 mg/mL Hydrocortisone, 100 ng/mL Cholera toxin and 10 µ/mL insulin. Media glucose concentrations modeling hyperglycemia (4.5 g/L) or normoglycemia (1.0 g/L) were followed to synchronize with reported media compositions [52]. We validated all cell lines employing short tandem repeat analyses using the PowerPlex^®^16 system (Promega).

### Chemical synthesis and chemoenzymatic labeling

Chemical probes were synthesized according to published reports, including O-GlcNAcase (OGA) inhibitor Thiamet-G [53] and azide labeled O-GlcNAc metabolic labeling sugar Ac_4_GalNAz [54]. Probes were synthesized, purified, and characterized using ^1^H-NMR, ^13^C-NMR, and mass spectrometry to match reported spectra. OGT inhibitor OSMI-4 [55] was originally provided by the Suzanne Walker group at Harvard Medical School, and then purchased from GLP Bioscience (Cat# GC31517). Inhibitors were solubilized in DMSO. All lysis buffers included OGA inhibitor (10 µM thiamet-G) to sustain O-GlcNAc levels. Click-IT^™^ O-GlcNAc enzymatic labeling kit (#C33368) was obtained from ThermoFisher. Biotin-alkyne (#1266-5) and TAMRA-alkyne (#1255-5) were purchased from Click Chemistry Tools (Scottsdale, AZ). For chemoenzymatic O-GlcNAc labeling, cells were cultured in low (1.0 g/L) or high (4.5 g/L) glucose media for 72 h then lysed in RIPA buffer (ThermoFisher #89900) with sonication using a QSonica Q500 ultrasonic processor equipped with a water bath sonic cup horn (Fisher Scientific FB-431C2). Protein was quantified and 200 µg total protein per sample were treated by addition of 600 µL of cold methanol followed by 150 µL of cold chloroform. Samples were vortexed for 15 s before 400 µL of cold milli-Q water was added to precipitate the protein. O-GlcNAc Chemoenzymatic labeling using the Click-IT^™^ kit were carried out as recommended by the manufacturer (ThermoFisher, #C33368). The azide labelled lysates were reacted with either TAMRA-alkyne or biotin-alkyne using the copper catalyzed azide-alkyne cycloaddition (CuAAC) click chemistry reaction. Briefly, azide labelled samples were resuspended in 200 µL of 1% NP-40 buffer and sonicated for total dissolution. The click reaction cocktail was made by mixing 5 µL of 2 mM TAMRA-alkyne (or biotin-alkyne), 2 µL of 10 mM Tris((1-benzyl-4-triazolyl-) methyl) amine (TBTA) and 4 µL of freshly made 50 mM copper (II) sulphate pentahydrate (CuSO_4_•H_2_O) and gently vortexed for 10 s before the addition of 4 µL of freshly prepared 100 mM sodium ascorbate solution in water and vortexed for additional 10 s. The 15 µL cocktail was then added to a 1.5 mL centrifuge tube containing the proteins, mounted on a Labquake^™^ end-over-end rotary mixer, and incubated at lab temperature (approximately 25 ^o^C) for 30 min. Proteins were precipitated as described above and washed with additional round of 450 µL cold methanol to remove excess alkyne reagent. The samples were then resuspended in 40 µL 1% SDS in 20 mM HEPES (pH 7.9) and sonicated for total dissolution in the buffer. To visualize samples, 20 µL of each sample was run on SDS-PAGE and visualized on fluorescence channel (for TAMRA-labelled samples) or immunoblotted with streptavidin-HRP (for biotin-labelled samples) using an iBright^™^ FL-1500 imaging system (ThermoFisher). For immunoprecipitation and protein-specific immunoblotting, biotin-labelled samples were resuspended in 200 µL NP-40 buffer and enriched with 30 µL of streptavidin coated magnetic beads (NEBS, #S1420S) for 1 h at 4 ^o^C with gentle rotation. The resin was separated with a magnetic rack, the supernatant was collected, and the resin was washed three times with Immunoprecipitation Buffer (IP Buffer, Pierce #87787). O-GlcNAcylated proteins were eluted with 20 µL of 1X sample buffer (ThermoFisher #NP0007), 3 µL of 10X reducing agent (ThermoFisher #NP0004), and 7 µL of IP buffer for SDS-PAGE and immunoblotting for TET1. Due to a lack of validated O-GlcNAc standards, 200 µg of total protein was used for each labeling reaction as input loading control.

### Immunoblotting and cell labeling

Western blotting procedures were carried out following a standard protocol. Briefly, cells were allowed to reach 80–90% confluency and collected using RIPA buffer (ThermoFisher, #89900) or 1% NP-40 buffer (150 mM NaCl, 50 mM Tris-Cl (pH 7.4), 1% NP-40) supplemented with Roche cOmplete^™^ protease inhibitor cocktail (#11,836,170,001). Protein lysates from OSMI-4-treated cells were collected after 72 h of probe treatment. Cell lysates were clarified by centrifugation at 17,000 x g for 15 min at 4 ^o^C. The supernatant containing soluble protein fractions were collected and quantified using Pierce^™^ Rapid Gold BCA Protein Assay kit (#A53225) and analyzed by SDS-PAGE and protein specific antibody western blot. Antibodies used for Western blot are: Anti-OGT (Cell Signaling Technology (CST), #24083), Anti-O-GlcNAc CTD110.6 (CST, #9875S), Anti-O-GlcNAc MultiMAb (CST #82332) Anti-Actin (Sigma, #A3853), Anti-Tubulin (Invitrogen, #MA1-80017), Anti-TET1 (Abnova Taiwan, #H00080312), Anti-TARDBP (Abnova, #H000234350-M01). Full blots and gel images are shown in the Additional file 1: Online Resources. Flow cytometry was conducted on a BD Biosciences LSR II instrument. During flow cytometry, the specified GFP-overexpressing MDA-MB-468 line or MBD2v2-overexpressing MDA-MB-468 line [12] were incubated with fluorochrome-labeled monoclonal antibodies against human CD44 (PE-Cyanine7, eBioscience Catalog # 25-0441-82), CD133 (APC, Miltenyi Biotec Catalog # 130-113-668) and EpCAM (PE-conjugate, BD Biosciences Catalog #566841) proteins following our previous method [34]. Cells were treated with DMSO or OSMI-4 OGT inhibitor for 48 h prior to FACS analysis. Gating strategies and all data are presented in Additional file 1: Online Resource 4.

### Quantitative real-time RT-PCR (qRT-PCR)

RNA was isolated from cultured and treated cell lines or from pulverized snap-frozen tumor tissue using the Qiagen RNeasy kit following the manufacturer’s procedure. mRNA samples were collected from OSMI-4-treated cells after 24 h. The RNA was converted to cDNA using the High-Capacity RNA-to-cDNA kit (ThermoFisher). The cDNA was diluted to 50 ng/µL working concentration for all samples and 2 µL of the working cDNA samples were then combined with SYBR Green PCR Master Mix reagents (ThermoFisher) to make up 20 µL reaction volumes in 96-wells plates. qRT-PCR reactions were run in three technical replicates using StepOnePlus Real-Time PCR System (ThermoFisher). mRNA expression of actin and/or PUM1 were used as control for all qRT-PCR experiments and the expression data were calculated using the delta-delta Ct method. All primers were purchased from Integrated DNA Technologies Inc. (Coralville, IA) or Fisher Scientific (TaqMan) and used as described previously [32, 33]. All primer sequences or Taqman catalog numbers are found in the Additional file 1: Online Resources.

### TET activity assay

MDA-MB-231 cells originally grown in high glucose (4.5 g/L) media was subcultured in low glucose (1.0 g/L) media for 72 h. Nuclear protein lysates were collected from both High and Low glucose media cultured cells using NE-PER nuclear and cytoplasmic extraction reagents (ThermoFisher #78833) supplemented with cOmplete mini EDTA free protease inhibitor cocktail (Roche #11836170001. Protein lysates were quantified by BCA protein assay using Pierce Rapid Gold BCA protein assay kit (ThermoFisher #A53226). TET activity assay was carried out using TET hydroxylase activity quantification kit (Fluorometric) (Abcam #Ab156913) following the manufacturer’s protocol and 5 µg nuclear protein lysates per sample. We also observed that protein lysates older than 48 h (even stored at −80 °C) gave lower TET activities. A wait time of up to 30 min was required to observe color development following the addition of the final reagent prior to fluorescence measurement.

### Transient knockdown

For OGT knockdown, we transfected ON-TARGET plus SMART pool human OGT siRNA (Dharmacon^™^ # L-019111-00-0005), TET1 (L-014635-03-0005), and TARDBP (L-012394-00-0005) versus ON-TARGET control pool non-targeting pool siRNA (#D001810-10-05) as control using DharmaFECT^™^ transfection reagent as described by the manufacturer. The SMART pool is a combination of 4 different siRNA oligos optimized for knockdown in human cell lines, which we used here in place of two distinct siRNA sequences for OGT knockdown and its corresponding SMART pool control knockdown.

### Transient TET1 overexpression in TNBC cell line

MDA-MB-231 cells were cultured in high glucose (4.5 g/L) DMEM media supplemented with 10% FBS and 1% Pen/Strep was seeded in 6-wells plate at a density of 1.5 × 10^5^ and incubated overnight. Recombinant adenoviral constructs, Ad-h-TET1 (RefSeq#: NM_030625) or Ad-GFP (Lot#: 20220823T#21) were purchased from Vector Biolabs (Vector Biolabs Inc., PA, USA). The viral stocks were diluted in media containing reduced serum (2% FBS) and 1 mL of the Adenoviral media was added to each well to make the MOI = 50 TU/cell. The adenoviral media was replaced with fresh media containing 10% FBS after 12 h. Media was replaced with fresh media containing 10 μm OSMI-4 24 hours post infection. mRNA was collected for qRT-PCR 24 h post-OSMI-4 treatment, which followed 24 h of adenovirus treatment. Protein extracts were collected for immunoblotting 24 h post-OSMI-4 treatment, which followed 48 h of adenovirus treatment.

### Stable overexpression of MBD2_V2 and GFP in MDA-MB-468 cells

MDA-MB-468 cells were transduced with packaged lentiviral particles purchased from Cyagen Biosciences (Santa Clara, CA) following our reported procedure [32, 33]. Both human MBD2c (NM015832.4) and GFP genes were subcloned into a lentiviral expression vector downstream of a CMV promoter and the construct sequenced to verify the accuracy of the sequence orientation. The transduced cells were then selected with 1.0 µg/mL puromycin. Following visual florescence image observation of GFP protein expression, both the MBD2_v2 and GFP expression levels were detected at mRNA and protein levels through qRT-PCR and western blotting [32].

### Animal work

All experiments and procedures involving animals and their care were pre-reviewed and approved by the Wayne State University Institutional Animal Care and Use Committee. All tumor samples analyzed in this work had been previously prepared during data collection for the prior study [12]. Briefly, female B6.129S7-Rag1tm1Mom/J (B6.Rag1-/-) mice were purchased from Jackson Laboratory (cat. no. 002216) at 5-weeks old and were acclimated for 1 week on standard chow diet. After 1 week, all mice were switched to and thereafter maintained on a purified diet. For experiments assessing tumor formation without consideration of diet-induced obesity (DIO), mice were fed a control formula (kcal fat% = 10, gram% = 4.3) from Research Diets (cat. no. D12450B). To study the effects of DIO, groups of mice were randomized to receive the control formula diet or a high-fat matched formula (kcal fat% = 60, gram% = 35, Research Diets, cat. no. D12492). At 11 weeks old (5 weeks of formula diet), mice were aseptically inoculated with cancer cells, in the flank, subcutaneously by injection using a 1 cc TB syringe with a 25 g half-inch needle, in a volume of 0.1–0.25 mL with matrigel (1:1 ratio). The resulting tumor masses (mg) were calculated based on caliper measurements. In experiments assessing effects of DIO on MDA-MB-468 tumor formation, mice were inoculated with a titer of 10^6^ cells (unilaterally). For these experiments there were 6 inoculations per group (12 mice total). To assess DIO-induced OGT levels, tumor RNA was extracted as described above from snap-frozen tissue. To assess DIO-induced O-GlcNAc levels, tumor proteins were extracted from snap-frozen tumors using the NE-PER Nuclear and Cytoplasmic Extraction Kit (cat. no. 78833, Thermo Fisher Scientific) and immunoblots carried out as described above.

### Statistical analysis and bioinformatics

Statistical analysis was performed using Bioconductor R 3.3.2 and GraphPad Prism. Graphs were generated with GraphPad Prism. *P* values ≤ 0.05 are reported as significant and *P ≤ 0.05, **P < 0.01, ***P < 0.001 indicates level of significance. Linear regression or Student’s t-test (two-sided) were applied when two conditions were compared, including qRT-PCR analysis. For analysis of 3 or more experiment conditions, a mixed-model approach was applied. To identify significant differences among gene expression data from The Cancer Genome Atlas (TCGA), analysis of variance (ANOVA) with Tukey’s HSD test was performed. For analysis of gene expression according to intrinsic subtyping, microarray data and PAM50 subtype annotation from TCGA were downloaded via cBioPortal. For analysis of clinically relevant subtypes, RNA-sequencing data from TCGA were downloaded from The National Cancer Institute Genomic Data Commons data portal. Non-tumor samples, N = 8; TNBC/basal-like samples, N = 81; HER2-enriched, N = 53; luminal A, N = 208; luminal B, N = 110.

## Results

### OGT mRNA is overexpressed in tumors from TNBC patients and activates TET1 activity in TNBC cell line

**Fig. 2:**
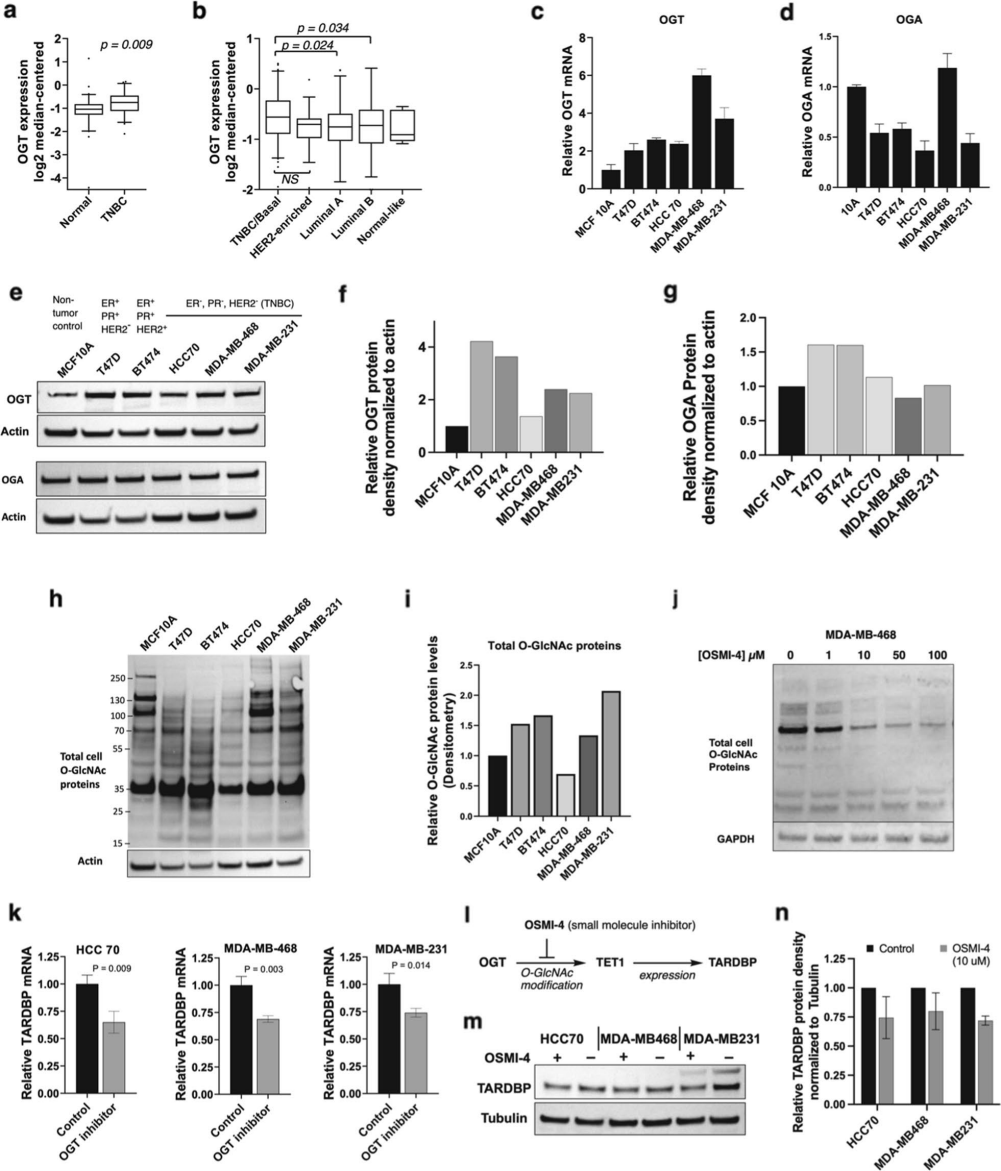
OGT is upregulated in TNBC tumors and cell lines. **a**, **b** Whole genome expression analyses of human patient samples targeting OGT mRNA expression in TNBC, matched breast tissue, and other subtypes of breast cancer. Line equals median, means were compared. Non-tumor samples, N = 8; TNBC/basal-like samples, N = 81; HER2-enriched, N = 53; luminal A, N = 208; luminal B, N = 110. NS indicates no significance (p > 0.05). **c**, **d** qRT-PCR analysis of OGT and OGA in non-tumor breast (MCF 10 A), two hormone-expressing BC lines, and three TNBC cell lines. Bars indicate standard error of the mean (SEM) from 3 independent experiments. **e** Western blot analysis of OGT and OGA protein expression in non-tumor MCF 10 A and tumor lines, with hormone status indicated. **f**, **g** Densitometry analysis of OGT and OGA expression in 6 cell lines, displayed relative to the MCF10A OGT band and normalized to actin. N = 1. **h** Western blot analysis of total cell O-GlcNAc (CST O-GlcNAc MultiMAb antibody) in 6 breast cell lines. N = 1. **i** Densitometry for total O-GlcNAc levels in 6 breast cell lines. **j** Dose-response for total cellular O-GlcNAcylation with OSMI-4 in MDA-MB-468 cells, 48 h treatment. Western blot performed with anti-O-GlcNAc MultiMAb. Bars indicate SEM of 3 biological replicates, with 3 technical replicates for each cell line. **k** TARDBP mRNA analysis by qRT-PCR upon OGT inhibition with OSMI-4- (10 µM, 24 h in low glucose media). Data is normalized to vehicle-treated controls. **l** Hypothesis for OGT inhibition impact on the TET1-driven target gene TARDBP based on published TET1 pathway [ref. 13]. **m** Western blot analysis of TARDBP protein expression in OSMI-4 treated (10 µM, 48 h in low glucose (1.0 g/L) media) or control (DMSO) treated TNBC cell lines. N = 2. **n** Densitometry analysis of TARDBP expression with OSMI-4 treatment, displayed relative to the untreated cells and normalized to tubulin

O-GlcNAc transferase (OGT) is the single gene product responsible for all cellular O-linked modifications of protein serine/threonine residues with the monosaccharide N-acetylglucosamine (GlcNAc) [56]. OGT is an essential gene in all cell types, underscored by it being the least frequently mutated glycosyltransferase in humans [47]. Despite the fact that OGT expression levels are normally tightly regulated in human cells [57], our analysis of patient data in The Cancer Genome Atlas (TCGA) found significant elevation of OGT in TNBC tumors over other breast cancer subtypes and non-tumor tissue. OGT was significantly overexpressed in TNBC patient tumors (Fig. 2a) versus matched normal tissue. Between subtypes of breast cancer patient tumors, TNBC/basal tumors showed significantly higher OGT expression than estrogen/progesterone receptor-expressing luminal A/B breast tumors (Fig. 2b). We performed the same analysis for the single human enzyme that removes O-GlcNAc, the O-GlcNAcase (OGA). As shown in Additional file 1: Online Resource 1, OGA was not significantly overexpressed in any BC subtypes relative to normal tissue, but OGA expression was significantly higher in luminal A and B compared to TNBC/basal patient samples. High OGT and low OGA expression in patient tumors suggest that TNBC subtypes were expected to display the highest levels of O-GlcNAcylation among the BC subtypes evaluated.

We analyzed OGT and OGA levels in a panel of BC cell lines, including three TNBC cell lines—HCC 70, MDA-MB-468, MDA-MB-231—as well as luminal cell model T47D and HER2-overexpressing model BT474 lines. For comparison, we profiled the non-cancer breast epithelial cell line MCF 10 A. We used qRT-PCR to characterize mRNA levels in TNBC (Fig. 2c, d) and immunoblotting to characterize protein levels (Fig. 2e**)**. In all tumor types, OGT was overexpressed at the protein level, matching prior reports [49–51]. We focused on the TNBC subtypes because TNBC lines are differentially selective to OGT inhibition compared to hormone-positive BC models, suggesting increased reliance on OGT in TNBC subtypes [51]. We observed between 2-and 6-fold increased basal expression of OGT in the three TNBC lines in our panel (Fig. 2c), which in all cases revealed upregulated OGT levels compared to the non-tumor line MCF 10 A. Though we saw high levels of OGT in the luminal A/B model T47D, high concurrent OGA levels may serve to normalize overall O-GlcNAc levels compared to the TNBC lines (Fig. 2h, i). Altogether, we observe distinct patterns of O-GlcNAc between the different cell types, with the TNBC cell lines displaying an emphasis on higher molecular weight protein O-GlcNAc bands (Fig. 2h). We previously reported significant elevation of pathway genes TET1, TARDBP, and SRSF2 in TNBC tumor samples obtained from obese (BMI > 30) compared to non-obese (BMI < 30) patients, as measured by qRT-PCR [32]. TCGA analysis of TET1, TARDBP, and SRSF2 is presented in Additional file 1: Online Resource 1. Profiles of OGT, OGA, TET1, TARDBP, and SRSF2 in patient tumors are consistent with an OGT-TET1-TARDBP-SRSF2 regulatory link in TNBC. Patient expression patterns directed our focus on TNBC to determine O-GlcNAc-driven effects of a CSC pathway.

Our next goal was to map how OGT leads to effects in CSCs [50]. We hypothesized that the robust, known biophysical interaction of OGT with TET1 [35, 37, 58] could impact our TET1-driven pathway toward CSC expansion in TNBC [12, 32, 33]. The OGT inhibitor OSMI-4 [55] showed dose-dependent inhibition of cellular O-GlcNAcylation using a global antibody against O-GlcNAc modifications (Fig. 2j). If OGT is upstream of this TET1 pathway, then OGT inhibition should show a reduced expression of TET1 target gene TAR DNA-binding protein (TARDBP) (Fig. 2l). Using three TNBC cell lines—HCC 70, MDA-MB-468, and MDA-MB-231—our data demonstrate that TARDBP mRNA levels were significantly reduced upon OGT inhibition (Fig. 2k–n). OGT inhibition led to ca. 30% lower TARDBP levels, suggesting that O-GlcNAcylation of TET1 was an activating modification for TET1-driven gene expression. Due to the myriad ways which O-GlcNAc levels alter cell functions, we next conducted a series of selective experiments to verify that OGT-catalyzed O-GlcNAc modification of TET1 occurs in TNBC cells.

### Hyperglycemic conditions activate TET1 through OGT-catalyzed O-GlcNAc glycosylation

**Fig. 3:**
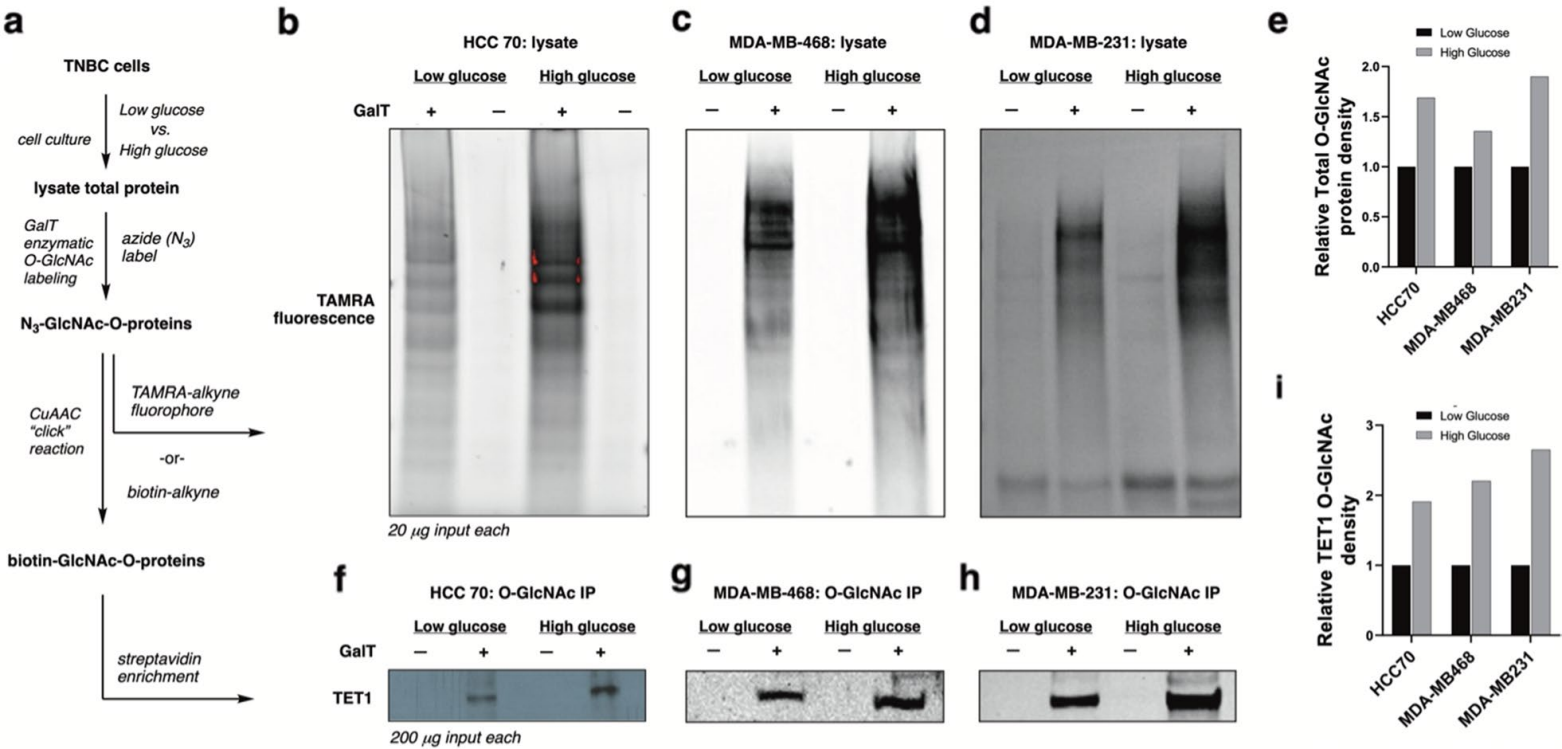
Chemical labeling of O-GlcNAcylated proteins in TNBC cells. **a** Scheme for O-GlcNAc detection: chemoenzymatic labeling using GalT (N-acetylgalactosaminyl transferase mutant Y189L) and copper-catalyzed azide-alkyne cycloaddition (CuAAC) click chemistry. **b**–**d** Total cell O-GlcNAcylation measured with TAMRA-red fluorophore-alkyne click reaction. N = 2, representative images shown. Cells were maintained in High glucose = 4.5 g/L or Low Glucose = 1.0 g/L DMEM media for at least 3 days prior to analysis. **e** Band densitometry for total O-GlcNAc staining, presented as total band density of high/low glucose. **f–h** O-GlcNAc enrichment using biotin click labeling and streptavidin beads followed by TET1 immunoblot. N = 2, representative images shown. **i** Band densitometry for TET1 O-GlcNAc labeling, presented as total band density of high/low glucose

Glucose flux leads to increased O-GlcNAcylation via the hexosamine biosynthetic pathway [41], offering a potential connection between hyperglycemia and TET1 target gene expression. We used a chemical strategy to label O-GlcNAc modifications on proteins in TNBC cells (Fig. 3a) to determine whether glucose flux drove TET1 O-GlcNAcylation by OGT. Briefly, cell lysates were treated with an engineered galactosyltransferase enzyme (GalT) that selectively installs an azide chemical label directly onto O-GlcNAc protein modifications [59, 60]. The resulting azide-labeled O-GlcNAcylated proteins (N_3_-GlcNAc-O-proteins) were reacted with labels using “click” chemistry, an irreversible copper-catalyzed azide-alkyne cycloaddition (CuAAC) reaction between the O-GlcNAc-azide species and the fluorescent TAMRA-alkyne reagent. These chemical biology labeling reactions revealed the total O-GlcNAcylation patterns in TNBC cells, which were compared between conditions using the total TAMRA fluorescence counts (Fig. 3b–e). Culturing three TNBC lines in low glucose (1 g/L) vs. high glucose (4.5 g/L) media revealed that hyperglycemic conditions elevated total O-GlcNAc modification levels in all three cell lines by ca. 1.3-2 fold.

We next replaced the fluorescent TAMRA-alkyne click reaction reagent with biotin-alkyne to enable affinity purification via biotin-streptavidin immunoprecipitation (IP). We used biotin-alkyne click reactions to install biotin on all cellular O-GlcNAc proteins. Biotin-labeled glycoproteins were enriched on streptavidin beads, then probed using western blot for TET1-specific bands. We used this biotin click/streptavidin enrichment/TET1 immunoblot sequence to verify that TET1 was O-GlcNAc modified in all three TNBC cell lines (Fig. 3f–i). Furthermore, cells grown in high glucose showed ca. 1.8–2.6 fold higher TET1-O-GlcNAc enrichment compared to low glucose samples (Fig. 3f–i). To normalize protein loading for each labeling reaction, we used identical total protein input (200 µg) as the loading control for our IP conditions due to a lack of validated O-GlcNAc standards. As a final confirmation of TET1 O-GlcNAcylation, we overexpressed a Flag-HA epitope tagged-TET1 catalytic domain and used metabolic engineering/labeling to install a clickable O-GlcNAc chemical reporter on TET1 in HEK293T cells (Additional file 1: Online Resource 2) [54]. When Flag-HA-TET1-expressing cells were fed azide-labeled sugar analogs, CuAAC click reaction confirmed that the O-GlcNAc modification of TET1 occurred in living cells (Additional file 1: Online Resource 2).

We additionally tested the impact of short-term glucose treatment on overall OGT levels in the TNBC lines under study. OGT mRNA levels in low vs. high glucose were found to be unchanged or even slightly reduced upon switching to high glucose media (Additional file 1: Online Resource 3), which matches prior reports whereby acute hypoglycemia increases OGT expression to rescue the lowered hexosamine biosynthetic pathway flux [61]. These data suggest that acute hyperglycemia enhanced TET1 O-GlcNAcylation through increased OGT activity rather than elevated OGT expression patterns.

**Fig. 4:**
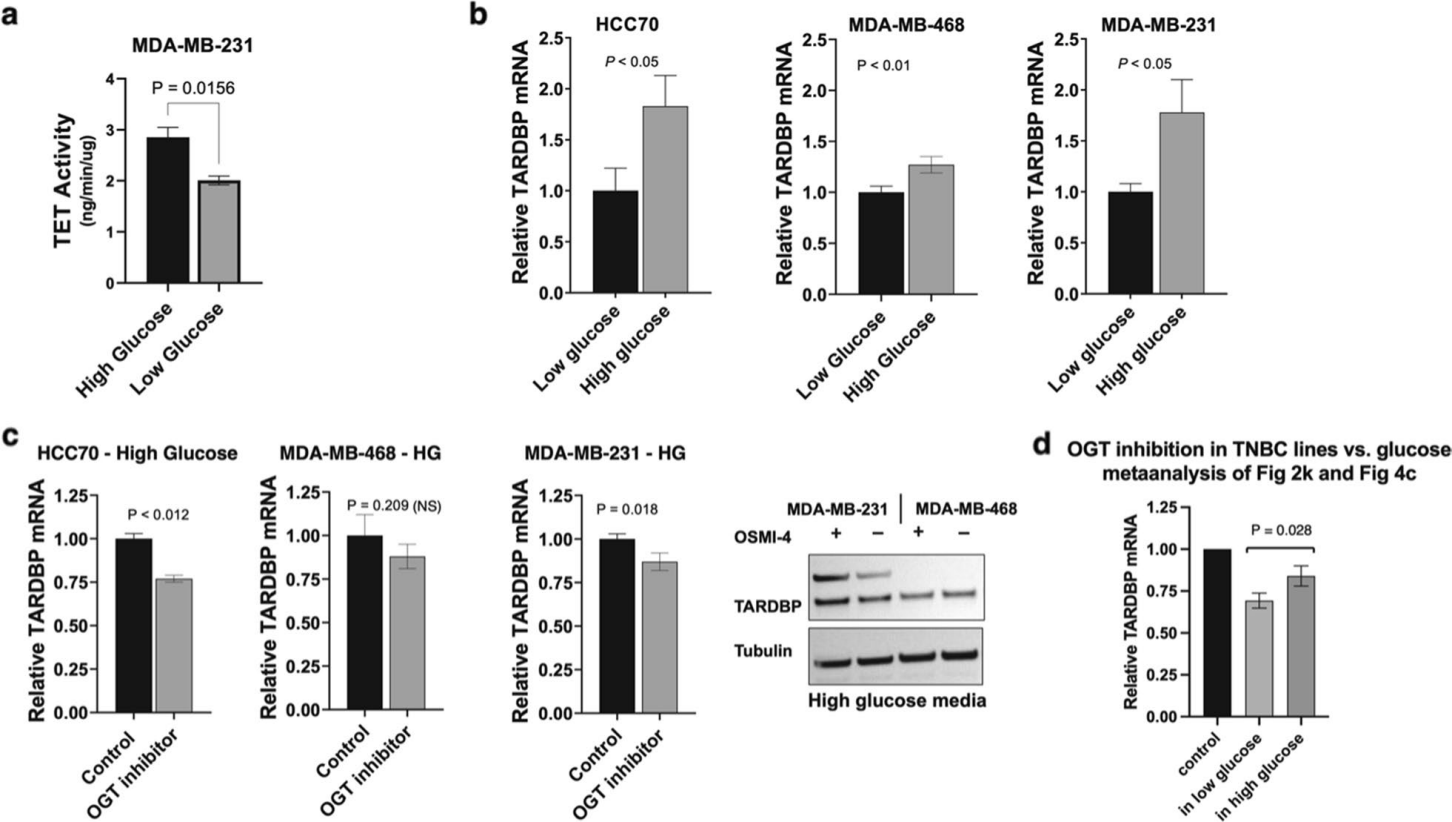
Glucose effects of TET1 activation and OGT inhibition in TNBC lines. **a** Comparison of total cellular TET activity using a 5-hydroxymethyl cytosine (5hmC) assay from TNBC cells grown in low vs. high glucose. MDA-MB-231 cells were cultured in low glucose (1.0 g/L) or high glucose (4.5 g/L) media for 3 days prior to nuclear extraction and 5hmC analysis. Bars indicate SEM of 3 technical replicates per condition, with 3 biological replicates in MDA-MB-231 cells. **b** Comparison of TARDBP mRNA levels by qRT-PCR between high and low glucose conditions. Media containing high glucose (4.5 g/L) was added for 24 h following growth in low glucose media (1.0 g/L). **b** TARDBP mRNA levels in OSMI-4 treated (10 µM, 24 h) TNBC cells in high glucose conditions were measured by qRT-PCR, normalized to non-treated controls. Bars indicate SEM of 3 biological replicates, with 3 technical replicates for each cell line. **c** Comparison of the average OGT inhibition data between low (see Fig. 2k) and high glucose in three TNBC cell lines (HCC70, MDA-MB-468, and MDA-MB-231). **d** Western blot analysis of TARDBP with OSMI-4 treatment in high glucose media (N = 2, representative blot shown)

Our data revealed that glucose enhances OGT-catalyzed O-GlcNAcylation of TET1. Previous reports in murine embryonic stem cells (mESCs) confirm that O-GlcNAcylation of TET1 activates its enzymatic activity, catalyzing the formation of 5-hydroxymethylcytosine (5hmC) [37]. We previously reported that TET1 enzymatic activity is responsible for upregulating the expression of TARDBP using 5hmC pulldown of the TARDBP promoter as well as a 5hmC oligonucleotide competition assay to inhibit proteins that bind the TARDBP 5hmC promoter site [32]. In line with these reports, we predicted that hyperglycemia would activate the TET1 pathway via elevated OGT activity. Total cellular 5hmC levels increased in high glucose vs. low glucose conditions (Fig. 4a), which is a mixture of TET1, TET2, and TET3 activities. Consistent with evidence that OGT-catalyzed O-GlcNAcylation increases TET1 activity, we analyzed the expression levels of the TET1 target gene TARDBP. TARDBP expression levels increased ca. 1.3–1.8-fold when TNBC cells were exposed to high glucose growth media (Fig. 4b). We tested whether the OGT inhibitor OSMI-4 was active against the hyperglycemic TET1 activation. Indeed, cell lines responded to OGT inhibition using OSMI-4 (Fig. 4c). Intriguingly, 10 µM OSMI treatment under high glucose had noticeably reduced efficacy compared to 10 µM OSMI treatment in low glucose (data from Fig. 2). We averaged the inhibition across cell lines in low glucose (Fig. 2k) vs. high glucose (Fig. 4c) and noted glucose-based rescue of OGT inhibition (Fig. 4d). The reduced effect of OGT inhibition on TET1 activity suggested that very high glucose levels could partially rescue substrate-competitive OGT inhibition via increased UDP-GlcNAc substrate production, data that further supported the role of OGT catalytic function in TET1 activation.

Taken together, OGT inhibition and O-GlcNAc chemical biology labeling revealed that TET1 was an OGT substrate in TNBC cells. Furthermore, hyperglycemic conditions enhanced expression of the known TET1-regulated gene TARDBP and were affected via OGT-catalyzed O-GlcNAc modification, as judged by small molecule inhibition. It is known that O-GlcNAc modification regulates TET1 catalytic activity [37], so the data in Figs. 2 and 4 are in line with our hypothesis that cellular glucose levels affects O-GlcNAcylation levels, which in turn activates TET1 for TARDBP production. Based on these data, we hypothesized that suppressing OGT would inhibit our reported CSC-inducing tumorigenic pathway [32] because OGT’s catalytic activity regulated TET1 in TNBC cell lines.

### Pathway engineering implicated OGT in a TET1-based tumorigenic pathway

**Fig. 5:**
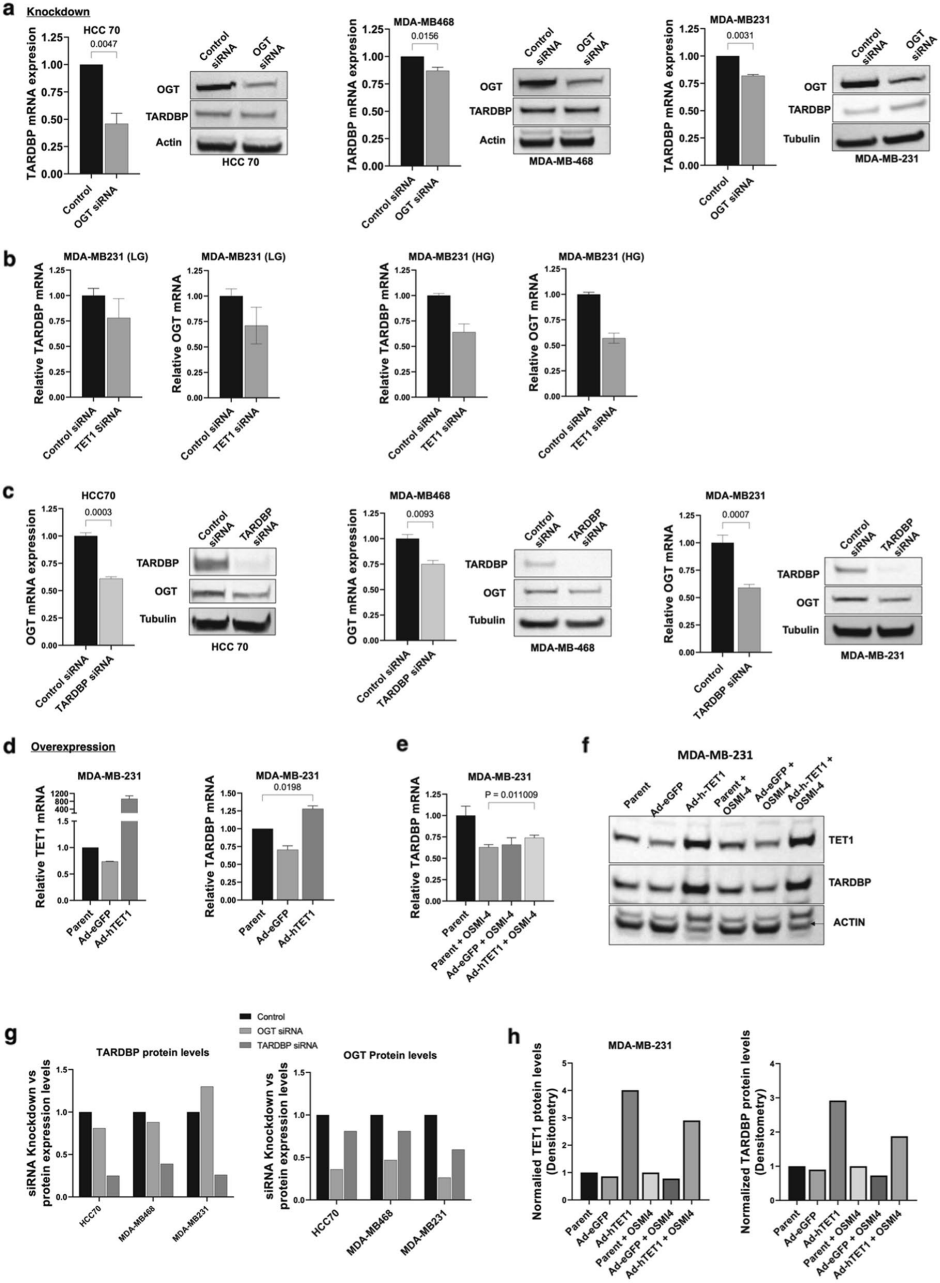
Knockdown and overexpression studies confirm the novel OGT pathway organization. **a** TNBC cells were treated with SmartPool OGT siRNA (4 targeting sequences) or a control, non-targeting SmartPool siRNA for 48 h. TARDBP mRNA and protein levels were measured. **b** TET1 knockdown with SmartPool siRNA (4 targeting sequences) in MDA-MB-231 cells in low glucose (LG, 1.0 g/L) or high glucose (HG, 4.5 g/L) media. Cells were maintained in each respective media for 3 days prior to knockdown. Bars indicate the SEM of 3 technical replicates, but a single biological replicate was performed so significance was not determined. **c** TNBC cells were treated with SmartPool TARDBP siRNA sequences and OGT mRNA and protein levels were measured. Data is normalized to non-silencing siRNA-treated controls. **d** Adenoviral vectors containing mRNA for GFP control or human TET1 were used to transiently overexpress the corresponding gene product for 24 h before mRNA was analyzed. Bars indicate the SEM of 3 technical replicates, 2 biological replicates performed. **e, f** TNBC cells overexpressing GFP or TET1 were treated with OSMI-4 for 24 h and the mRNA and protein levels of TET1 and TARDBP were analyzed. Bars indicate the SEM of 3 technical replicates, 2 biological replicates performed. **g** Densitometry analysis of TARDBP and OGT knockdown in three TNBC cell lines. Western blots are representative of at least 2 biological replicates per condition. All cells were maintained in low glucose = 1.0 g/L media for at least 3 days prior to analysis. **h** Densitometry analysis of overexpression-mediated TET1 and TARDBP immunoblot levels with OGT inhibition in MDA-MB-231 cells. Western blots are representative of at least 2 biological replicates per condition. All cells were maintained in low glucose (1.0 g/L) media for at least 3 days prior to analysis

We previously established that TET1 [32], SRSF2 [12], TARDBP [32], and MBD2_v2 [33] elements are all necessary for TET1-driven CSC reprogramming as part of a pathway (summarized in Fig. 1b) using knockdown and overexpression of each pathway component. Here, we used knockdown studies to engineer TNBC cell lines to verify OGT effects and regulation as a glucose-driven extension of this TET1 pathway leading to CSC maintenance. As expected, OGT knockdown recapitulated our results with the OGT inhibitor OSMI-4 (Fig. 5a). We observed significant TARDBP mRNA decrease but only a slight response at the protein level, perhaps due different timeframes of the siRNA effects on mRNA vs. protein levels in the 48 h knockdown timeframe. The aligned mRNA data from OGT knockdown and inhibitor treatment are consistent with our hypothesis that OGT protein expression, and specifically its catalytic activity, regulated TET1 because OSMI-4 is known to inhibit OGT catalytic activity through competitive substrate binding [55]. These results are supported by our data whereby O-GlcNAcylation and TET1 activity are each enhanced by glucose flux (Fig. 3).

We also engineered the expression of downstream pathway members and analyzed OGT expression levels. Knockdown of TET1 led to reduction of TARDBP mRNA (Fig. 5b), as expected [32]. Surprisingly, we observed reduced OGT mRNA levels in both low and high glucose upon TET1 knockdown, despite TET1 being “below” OGT in our proposed activation pathway (Fig. 5b). To confirm this finding, we additionally knocked down TARDBP and observed the same or greater reduction of OGT at both the mRNA and protein level in all three TNBC cell lines (Fig. 5c). These two knockdown studies revealed that TARDBP was necessary for high OGT expression levels. A known feature of OGT expression level regulation is via alternative splicing to produce mRNA encoding the functional gene product or, through a detained intron, an alternative transcript that will be rapidly degraded via nonsense-mediated mRNA decay [57]. Cells use detained intron splicing mechanisms to rapidly regulate OGT levels to maintain homeostasis. Because TARDBP is a DNA binding protein and a splicing modulator [62], we checked the SpliceAid database [63] and found a canonical TARDBP binding site on the OGT mRNA sequence (Additional file 1: Online Resource 4). A potential mechanism for TARDBP regulation of OGT, despite being downstream in the CSC pathway we proposed, was through OGT transcript stabilization via TARDBP’s predicted OGT mRNA binding activity [62]. We noted differences in the level of OGT reduction upon TARDBP knockdown between different cell lines, potentially reflecting differences in total cell OGT mRNA levels (see Fig. 1c, d). For example, MDA-MB-468 expressed the highest baseline levels of OGT (Fig. 1c) and responded least to OGT siRNA, but intriguingly showed stronger OGT loss upon TARDBP knockdown than in other TNBC cell lines.

The converse experiment of TET1 overexpression revealed that OGT inhibition can be partially resuced by more TET1 protein. We used an adenoviral vector to overexpress human TET1 vs. green fluorescent protein (GFP) as an adenovirus control. TET1 overexrpession led to higher levels of TARDBP (Fig. 5d). Upon addition of OGT inhibitor, TET1 overexpression partially rescued OGT inhibition effects on TARDBP production at the concentration used throughout this study (Fig. 5e, f). Figure 5g, h summarize the knockdown and overexpression immunoblotting results by densitometry, respectively. These data further support the OGT/TET1 pathway connection.

Upon OGT knockdown or inhibition (see Figs. 2 and 4, and Fig. 5), TARDBP mRNA and protein levels were reduced but not completely abolished. Our OGT suppression data is consistent with our hypothesis that OGT binds and activates TET1 activity via O-GlcNAcylation, as is observed in embryonic stem cells [35, 37]. OGT was not essential for baseline TET1 activity but rather served a regulatory (activating) role. Baseline TET1 activity is also known to be enhanced by O-GlcNAcylation from biochemical studies of recombinant TET1, OGT, and the OGT substrate UDP-GlcNAc [37]. The effect of TARDBP knockdown and OGT reduction suggests that the pathway we propose additionally includes a reciprocal element of OGT regulation by TARDBP, likely via mRNA binding (Additional file 1: Online Resource 4), though this hypothesis is yet to be experimentally tested. Our combined data suggest that OGT may raise TARDBP levels, which allows further elevation of OGT levels in a “feed-forward” manner (see Discussion, below).

**Fig. 6:**
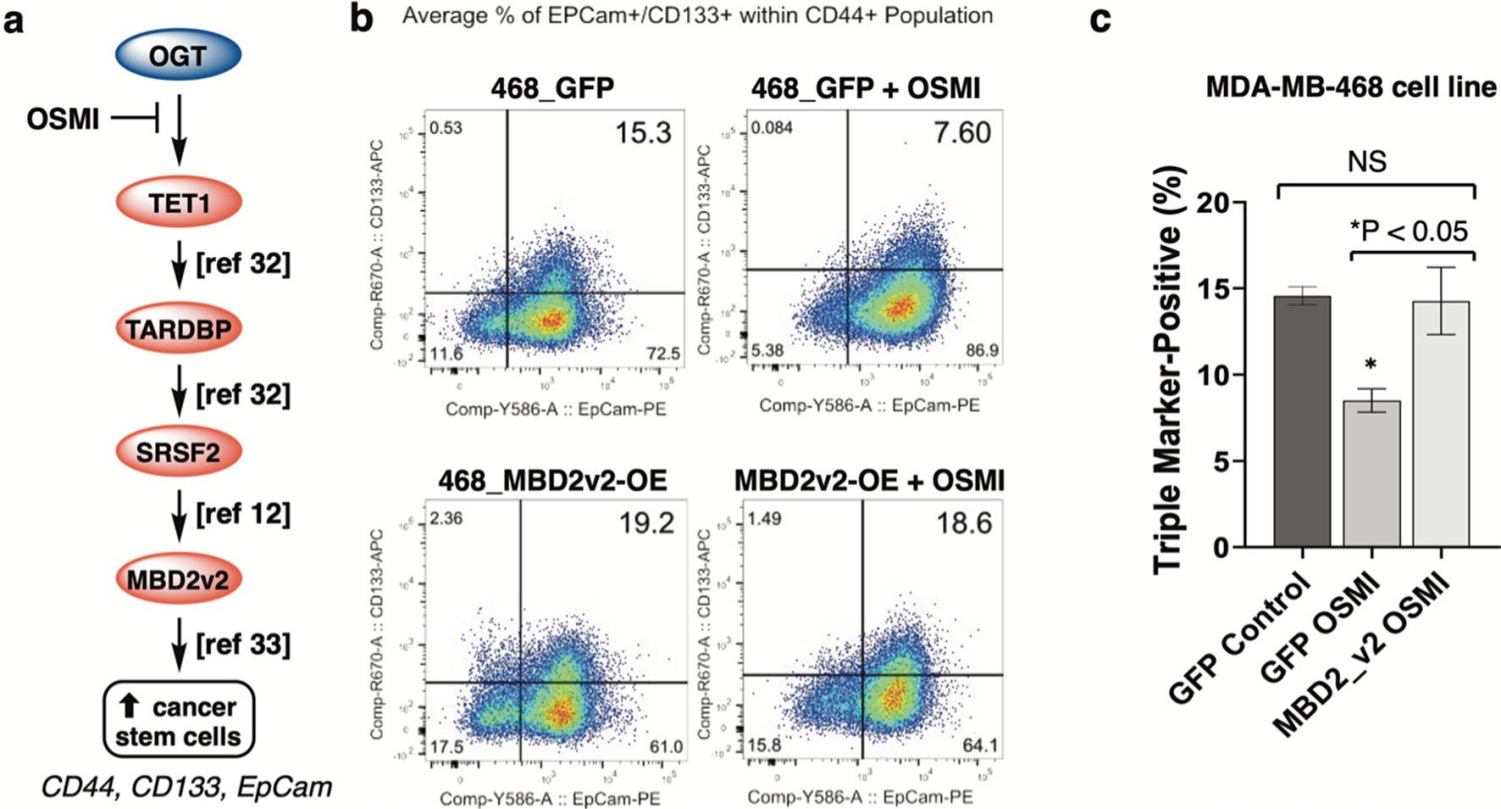
Impact of OGT inhibition on cancer stem cell maintenance in TNBC cells. **a** Hypothesized entry point for OGT to our published CSC expansion pathway [13]. **b** Fluorescence Activated Cell Sorting (FACS) analysis for triple stem cell marker expression under OGT inhibition. The CD44 + population was gated and analyzed further for CD133 and EpCam. Upper right quadrant indicates “triple positive” CSC cell populations. 468_GFP indicates control transfection cells (green fluorescent protein overexpression) and 468_MBD2v2-OE indicates stable MBD2_v2 overexpression as “rescue” construct. Representative data shown (full data and gating strategies shown in Additional file 1: Online Resource 5). **c** Analysis of FACS data. Lines indicate SEM, N = 3. OSMI treatment duration was 48 h prior to analysis, in low glucose media

To test whether TNBC cancer stem cell populations responded to OGT inhibition, we analyzed the production of stem cell markers in MBA-MB-468 cell lines [34]. We used fluorescence activated cell sorting (FACS) to analyze the population of CSCs using CD44, EpCam, and CD133 sorting as triple markers of cancer stem-like cells [29]. Cells that stained positive for these triple markers indicated CSCs that were distinct from bulk (non-CSC) TNBC cells. For this experiment, we grew TNBC cell line MBA-MB-468 that overexpressed GFP as a control or that overexpressed MBD2_v2 as a pathway protein downstream of TET1 and TARDBP (Fig. 6a). We chose MBD2_v2 as our rescue protein to avoid effects of TET1 vs. TET2 and TET3 compensation [64] as well as to avoid TARDBP-driven splicing effects that might affect OGT levels (Fig. 5). We hypothesized that GFP-TNBC cells would respond to OGT inhibition with reduced CSC levels, but MBD2_V2-TNBC cells would overexpress a downstream pathway member to rescue cells from OGT inhibition. Treatment of GFP-vector-overexpressing TNBC cells with OGT inhibitors significantly depleted the population of CSCs by almost 50% (Fig. 6b, c). The specific regulation of this pathway by OGT was confirmed by overexpression of MBD2_v2, an obesity related gene that is downstream of OGT in the CSC pathway presented here [12, 33]. MBD2_v2 overexpression was able to rescue OGT inhibition back to at least the same level as non-treated TNBC cells. Full flow cytometry data is shown in Additional file 1: Online Resource 5. Our data is consistent with our hypothesis that OGT’s catalytic activity is upstream of MBD2v2 during CSC expansion. When added to our previous studies on how SRSF2 regulates MBD2v2 [12] and, later, how TET1 activity affects SRSF2 via TARDBP production [32], our data with OGT and TET1 in the present study support that OGT is an upstream activator of this CSC pathway.

### TNBC tumor OGT and O-GlcNAcylation are elevated in animal models of hyperglycemic disease

**Fig. 7:**
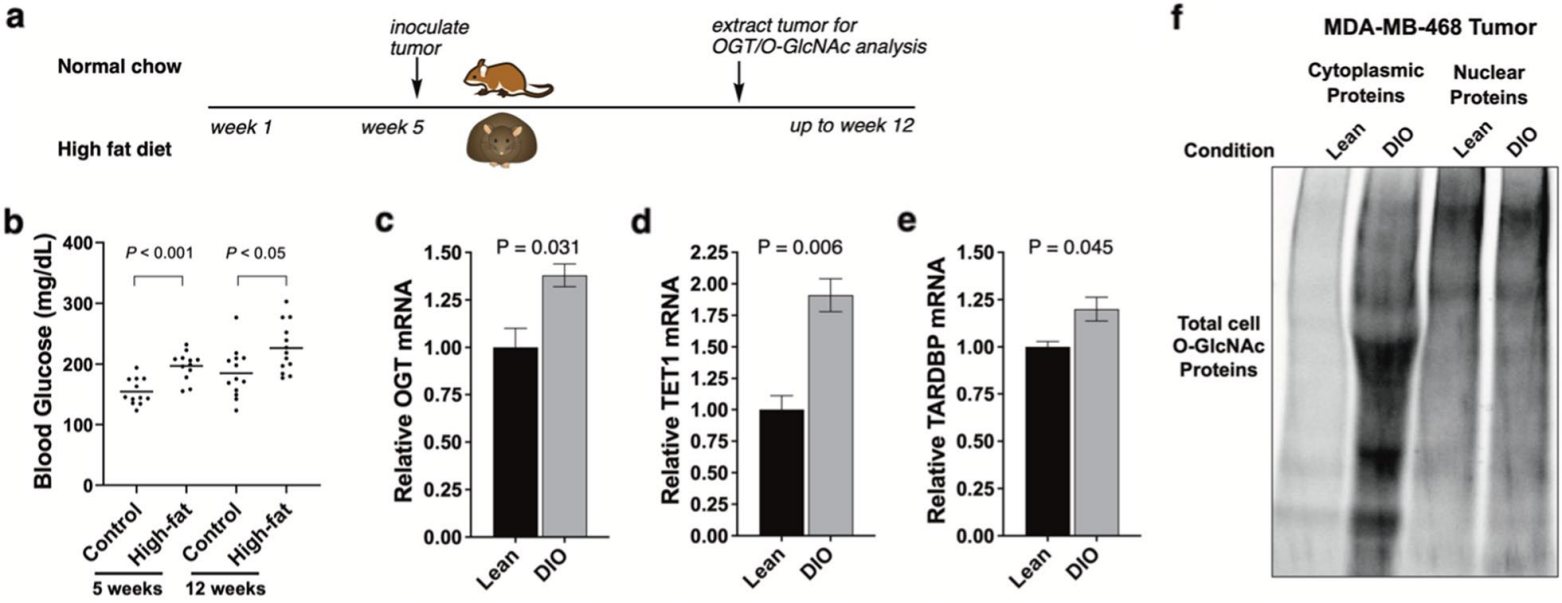
Obesity-associated hyperglycemia drives cellular OGT and O-GlcNAc levels in TNBC tumors. **a** Timeline for DIO induction and tumor studies. **b** Glucose in blood samples from control and high-fat diet at 5 and 12 weeks. 12 per group. **c–e** OGT, TET1, and TARDBP levels, respectively, in TNBC cell line MDA-MB-468-derived tumors harvested from DIO (N = 3) and lean control mice (N = 3). The qRT-PCR analysis of each sample was performed in triplicate and means normalized. **f** Chemoenzymatic labeling of total O-GlcNAc levels in TNBC tumors isolated from DIO vs. lean mice, N = 1. Mouse weight on diet is shown in Additional file 1: Online Resource 6

In our previous reports, we observe that diet-induced obesity (DIO) can upregulate pathway members TET1 and MBD2_v2 levels [12, 32]. To test the impact of a hyperglycemic tumor microenvironments on OGT, we used B6.Rag1 knockout mice, an obesity-compatible mouse model [65], to measure effects in TNBC tumors (Fig. 7a). Mice gained weight and attained hyperglycemia after 5 weeks on high fat diet (Fig. 7b and Additional file 1: Online Resource 6). We analyzed OGT, TET1, and TARDBP mRNA from TNBC tumors implanted in lean vs. DIO mouse models using qRT-PCR (Fig. 7c–e). We confirmed significant overexpression of all three pathway components in TNBC tumors grown in DIO mice vs. lean littermates. These data suggest that physiological hyperglycemia affected the pathway under study.

We also measured the impact of the obesity-associated hyperglycemic tumor microenvironment on O-GlcNAc levels in tumors grown in DIO vs. lean mice using chemoenzymatic labeling (Fig. 7f). Both cytosolic and nuclear fractions of a DIO-grown TNBC tumor compared with a lean TNBC tumor showed elevated overall O-GlcNAcylation, with the stronger effect observed on cytosolic O-GlcNAc proteins. We did not isolate enough tumor material to enable TET1 pulldown studies, which will be important to confirm our mechanism in future studies. We also only had one remaining sample to compare O-GlcNAc levels within (Fig. 7f, N = 1), so a larger study is currently underway. Our current data support our in vitro study of how high vs. low glucose cell culture conditions that elevate O-GlcNAc levels, which included TET1. The hyperglycemic aspect may contribute to additional OGT activity via hexosamine biosynthetic pathway flux from glucose to UDP-GlcNAc, the OGT substrate. Taken together, this data supports a yet-to-be confirmed hypothesis that obesity can elevate OGT expression and O-GlcNAcylation in tumors. We hypothesize based on our in vitro experimental results that in obese mice, elevated O-GlcNAc levels is a result of the OGT/TET1/TARDBP loop that feeds-forward to elevate OGT and O-GlcNAc level in hyperglycemic settings. We anticipate that OGT and O-GlcNAc are upregulated in TNBC tumors from obese relative to non-obese women, but that further analysis will require a study beyond the scope of this report.

### Discussion

Correlative studies between TNBC incidence and metabolic diseases including type 2 diabetes and obesity reveal a ca. 43–80% higher risk of TNBC [15, 27]. One aspect of this link could be metabolic reprograming in TNBC tumors [66]. OGT serves as a key nutrient and metabolism sensor in human cells because its substrate UDP-GlcNAc is glucose-derived via the hexosamine biosynthetic pathway [42]. Notably, OGT’s nutrient sensing role has been previously linked with breast cancer tumorigenesis via the transcription factor FoxM1, but since FoxM1 is not an OGT substrate there remains a missing link in this connection [49]. Establishing a mechanism for OGT-driven tumorigenesis would provide therapeutic insight into metabolic disease and TNBC risk. The reported physical interaction of OGT and TET1 [35, 37] led us to hypothesize that our recently reported TET1 pathway to TNBC tumorigenesis [12, 32] might be regulated by OGT.

**Fig. 8:**
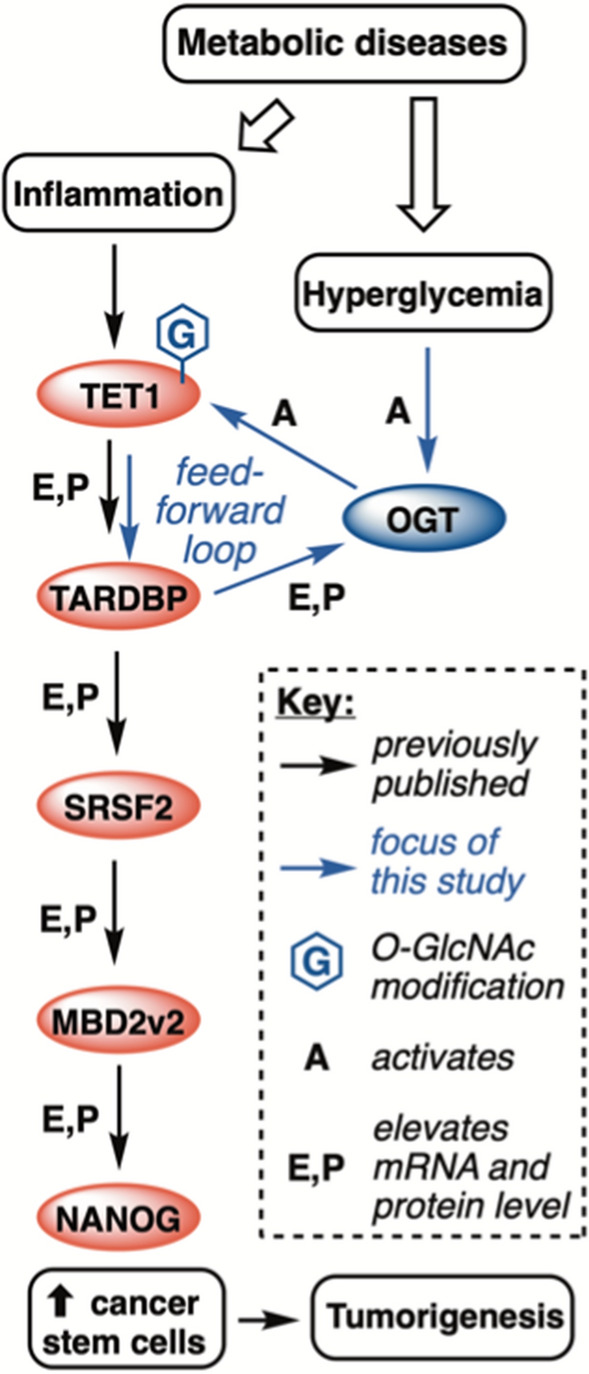
Working Model for the OGT-driven pathway presented in this study

Altogether, our data led us to propose a novel working model for OGT overexpression in TNBC tumors and its impact on a tumorigenic pathway (Fig. 8). We previously demonstrated that obese physiology fuels a pathway leading to enhanced TNBC tumorgenicity via activity of (TET1), which enables overexpression of TAR DNA-binding protein (TARDBP), a nucleotide binding protein. Elevated TARDBP activity positively regulates another splicing factor, serine and arginine rich splicing factor 2 (SRSF2). Higher TARDBP and SFRSF2 levels produce the tumorigenic splice variant 2 of methyl CpG-binding protein 2 (MBD2_v2) [12]. MBD2_v2 leads to the stem cell reprograming factor NANOG and CSC expansion [12, 32]. Notably, each member of the pathway has been implicated with TNBC tumorigenicity in vivo: MBD2_v2 and SRSF2 by our laboratory [12, 32, 33], NANOG by Thiagarajan et al., TARDBP by Ke et al. [67], and TET1 by Wu and colleagues.[68] Beyond breast cancer, OGT is increased as a biomarker for tumorigenesis, invasion, and poor prognosis in colon cancer [69].

Here, we conclusively demonstrated in TNBC model systems that OGT was an upstream regulator of this CSC pathway in a catalytic fashion; was itself regulated by splicing factors in this tumorigenic pathway; and that a key hallmark of metabolic disease, hyperglycemia, further activated this pathway. The TET1-centric pathway in our working model is also linked with inflammation, another major hallmark of obesity. In our prior study, we show that reactive oxygen species (ROS) generation via CoCl_2_ or IL-6 treatment enhances this TET1 pathway and ROS neutralization via catalase represses this pathway [32], potentially explaining the inflammatory hallmark of clinical obesity as a risk factor for TNBC [12]. Our new data suggests, potentially for the first time, that two distinct physiological hallmarks of obesity—systemic hyperglycemia and chronic inflammation—might converge on this TET1-driven pathway to drive cancer stem-like cell induction. Rescue of OGT inhibitor effects of cancer stem-like cell reprogramming was performed by MBD2_v2 overexpression, further solidifying the upstream regulatory placement of OGT in this pathway. Reinforcing these in vitro results, our models of TNBC tumors in DIO mice show elevated OGT levels, O-GlcNAc levels, and supported our published pathway whereby DIO mice display higher TNBC tumor initiation events than their lean littermates [12]. These data provide a possible mechanism for the correlation between metabolic disease patients and TNBC risk factors.

The unexpected regulation of OGT in this OGT->TET1->TARDBP pathway offers a potential mechanism to explain the higher levels of OGT mRNA that we observed in tumors between lean and DIO mice. In our model, upregulated tumor OGT levels could be a product of feed-forward regulation in three steps. In first step, obesity triggers systemic hyperglycemia, which activates TET1 to produce higher levels of the DNA binding protein TARDBP via increased OGT substrate levels. Second, TARDBP stabilizes or produces additional active OGT via splicing regulation—a known regulatory manifold for OGT [57]—to further activate TET1 for TARDBP production. Third, this process continues, resulting in the elevated OGT levels observed in TNBC patient data (Fig. 2a, b) and our DIO mouse data (Fig. 7c). This combined human cell line and animal data suggest that obesity in humans might similarly lead to elevated OGT levels in TNBC tumors. Indeed, our OGT analysis of The Cancer Genome Atlas breast cancer subtype dataset revealed that TNBC patients have higher OGT levels than other subtypes of breast cancer (Fig. 2a, b). If this feed-forward pathway for hyperglycemia-driven OGT activation is true, it might be applied to explain higher levels of OGT expression in other forms of cancer [70], including other non-TNBC subtypes of breast cancer, though we have not yet pursued this avenue of inquiry.

Inhibiting OGT might be one way to treat TNBC, as others have suggested [50, 51]. However, our OGT inhibition data for TARDBP production showed diminished effects at high glucose culture conditions compared to normal glucose conditions (Fig. 2k vs. Fig. 4c). Hyperglycemia leads to higher OGT substrate production (UDP-GlcNAc) in cells [41], and such elevated glucose to UDP-GlcNAc flux may outcompete the active site OGT inhibitor OSMI-4 in TNBC cells. If this finding is general, pre-clinical and clinical studies involving OGT may benefit from considerations of diet and physiology to account for elevated OGT levels and/or substrate availability in hyperglycemic settings. Also, effects of global OGT reduction might present untoward side effects because OGT is found in all mammalian cells [47]. The mechanism presented here suggests downstream targets, including TET1 [34] and TARDBP, as potential therapeutic alternatives to OGT inhibition that affect our proposed pathway in question with higher selectivity.

The studies conducted in this report indicated that hyperglycemia impacts CSC induction in TNBC via OGT-dependent glycosylation of TET1. However, there are several key limitations to this current study. Over 20 O-GlcNAc modification sites have been identified on murine TET1 [71], making it technically difficult to carry out site-specific mutation in TET1 to identify the key O-GlcNAc site(s) driving this pathway. Hyperglycemia is only one aspect of metabolic disease, so a more in-depth study of OGT-CSC pathway is needed using in vivo models that incorporate stable knockdown OGT/TNBC lines and/or OGT inhibitors with enhanced in vivo activity [72, 73]. Further studies are also needed to confirm that the OGT and O-GlcNAc effects we observed in obese versus lean mice are also at play in patient tumors of various body mass indices. Finally, the impact of OGT inhibition was partially reduced in TNBC cells exposed to hyperglycemic conditions. The reduced impact of the OGT inhibition in hyperglycemic settings is a potential impact on obesity/OGT inhibitor effectiveness that will bear further examination.

## Conclusions

The current report describes evidence to support that hyperglycemia drives OGT expression level and activity in a tumorigenic pathway identified in TNBC. We mapped OGT as an activating feature of the epigenetic regulator TET1. OGT was overexpressed in TNBC patients relative to luminal A/B subytpes of BC. In diet-induced obese mice, OGT expression and activity were increased relative to lean littermates and corresponded with higher levels of TET1-responsive cancer stem-like cell pathway member TARDBP. An interesting facet of this pathway was its feed-forward nature, whereby hyperglycemia led to enhanced activity of OGT, which in turn led to elevated OGT expression via the splicing factor TARDBP. Our initial data in mice suggested that OGT was upregulated in DIO animals compared to lean littermates, addressing one of the many missing mechanistic links connecting this metabolic disease with TNBC risk and progression. The majority of TNBC patients are obese or overweight [10, 14], increasing the relevance of this study toward determining a potential mechanism for the obesity-TNBC correlation. Because our data suggest that obesity and potentially other hyperglycemic diseases can both elevate OGT activity and drive a tumorigenic pathway in tumors, rising obesity rates [7] are concerning with respect to the pathway we put together here. We envision that continued studies of OGT and O-GlcNAc-associated mechanisms in will help address hyperglycemia-associated cancer risk as a critical unmet challenge in oncology.

## Supplementary Information

**Additional file 1:** **Online Resource 1.** Expanded TCGARNA-seq patient sample analysis for CSC pathway genes. **a**) O-GlcNAcaselevels between subtypes of breast cancer and normal tissue. **b–d** Pathway protein data and analysis, re-analyzed from data first analyzed andreported in our TET1 study.^1^ Non-tumor samples, N= 8; TNBC/basal-like samples, N = 81; HER2-enriched, N = 53; luminal A, N =208; luminal B, N = 110. **Online Resource 2.** Alternative confirmation that TET1 isO-GlcNAc modified. **a** Overexpression of Flag-HA-tagged TET1 catalytic domain in HEK293T and HEK293 cells. Stars indicate TET1-specific bands. **b** HEK293T cells were fed the azide-labeled per-acetyl galactosamineas a metabolic reporter of O-GlcNAc. Click chemistry was performed with biotinalkyne. HA immunoprecipitation was used to enrich Flag-HA-TET1. Streptavidinblot was used to visualize biotinylated samples. **Online Resource 3.** Comparison of OGT mRNA levels by qRT-PCRbetween high and low glucose conditions. Media containing high glucosewas added for 24 h following growth in low glucose media. Barsindicate variability across 3 technical replicates for each cell line. Data wascollected for 1 biological replicate per cell line, no significance wasdetermined. **OnlineResource 4.** SpliceAid diagram of TARDBP binding siteon OGT mRNA. Made using human OGT sequence andhttp://www.introni.it/splicing.html.**Online Resource 5a**. Gating strategy for analysis of cancer stem-like cell markers. Unstained controls for each condition were used to set the analysis parameters. Front scatter plotand side scatter plotwere use by areaor heightto determine cell morphology. CD44 was analyzed in the PE-Cy7 channel, CD133 was analyzed in the APC channel, and EpCam was analyzedin the Y780 channel to set background. The text below each plot indicates the sample type, then the gating strategy, and the number indicates the total number of events for each run. Cell lines: GFP-vector-expressing MDA-MB-468 cells or MBD2_V2-overexpressing MDA-MB-468 cell lines. **Online Resource 5b**. Analysis of cancer stem-like cell markers CD44, CD133, and EpCam in bulk TNBC cells. Cell lines: stable GFP-vector-expressing MDA-MB-468 cells were treated with DMSO control. Cells were first gated by CD44, then analyzed for CD133 and EpCam staining. All three markers represent cancer stem-like cells. Results presented for three biological replicates. **Online Resource 5c.** Analysis of cancer stem-like cell markers CD44, CD133, and EpCam in bulk TNBC cells. Cell lines: stable GFP-vector-expressing MDA-MB-468 cells were treated with OGT inhibitor. Cells were first gated by CD44, then analyzed for CD133 and EpCam staining. All three markers represent cancer stem-like cells. Results presented for three biological replicates. **Online Resource 5d.** Analysis of cancer stem-like cell markers CD44, CD133,and EpCam in bulk TNBC cells. Cell lines: stable MBD2v2-overexpressingMDA-MB-468 cells were treated with DMSO control. Cells were first gated byCD44, then analyzed for CD133 and EpCam staining. All three markers representcancer stem-like cells. Results presented for three biological replicates. **Online Resource 5e.** Analysis of cancer stem-like cell markers CD44, CD133,and EpCam in bulk TNBC cells. Cell lines: stable MBD2v2-overexpressing MDA-MB-468cells were treated with OGT inhibitor. Cells were firstgated by CD44, then analyzed for CD133 and EpCam staining. All three markersrepresent cancer stem-like cells. Results presented for three biologicalreplicates. **Online Resource 5f.** Analysis of cancer stem-like cell markers CD44, CD133,and EpCam in bulk TNBC cells. Bars indicate the standard error of the mean ofthree biological replicates. Statistical analysis: * indicates P ≤ 0.05, **indicates P ≤ 0.01. **Online Resource 6.** Weight of mice onhigh fat dietrelative to control diet. The difference was significant at 5 weeks, P < 0.001. 12 mice per group.

## Data Availability

The datasets generated and analyzed during the current study are available from the corresponding author on reasonable request.

## Abbreviations

TNBC: Triple-negative breast cancer
GlcNAc: N-acetylglucosamine,a sugar
O-GlcNAc: O-linked GlcNAc post translational modification on serine and threonine residues
UDP-GlcNAc: Uridine diphosphate N-acetylglucosamine, a sugar donor
OGT: O-GlcNAc transferase, adds O-GlcNAc to protein substrates
OGA: O-GlcNAcase, removes O-GlcNAc from protein substrates
CSC: Cancer stem-like cell
DIO: Diet-induced obesity
TET1: Tet methylcytosine dioxygenase 1
TARDBP: TAR DNA-binding protein 43, a splicing factor
SRSF2: Serine/arginine-rich splicing factor 2, a splicing factor
MBD2_v2: Methyl-CpG-binding domain protein 2, splice variant 2
NANOG: Homeobox protein NANOG, a stem cell reprograming transcription factor
GFP: Green fluorescent protein
CD44/CD133/EpCam: Triple protein markers for cancer stem-like cells
TCGA: The Cancer Genome Atlas (NIH program)
qRT-PCR: Quantitative reverse transcriptase polymerase chain reaction
“click” chemistry: Bioorthogonal reaction using cell- and protein-compatible motifs like azides and alkynes
CuAAC: Copper-catalyzed azide-alkyne cycloaddition reaction
GalT: Mutant β-1,4-galactosyltransferase (Gal-T1 (Y289L)) (enzyme that labels O-GlcNAc with an azide-sugar)
UDP-GalNAz: Uridine diphosphate N-acetylazidogalactosamine (click chemistry substrate for GalT)
Ac_4_GalNAz: 1,3,4,6-tetra-O-acetyl-2-N-acetylazidogalactosamine (metabolic labeling reagent)
TAMRA: 5-Carboxytetramethylrhodamine (red) fluorescent dye
OSMI-4: OGT small molecule inhibitor
