# Supplementary material for: Hyperglycemia and O-GlcNAc transferase activity drive a cancer stem cell pathway in triple-negative breast cancer
Source: Cancer Cell Int. 2023 May 25;23:102. doi: 10.1186/s12935-023-02942-6 (PMC10210312; doi:10.1186/s12935-023-02942-6)
Supplement: Supplementary file 1 — Additional file 1: Online Resource 1. Expanded TCGARNA-seq patient sample analysis for CSC pathway genes. a) O-GlcNAcaselevels between subtypes of breast cancer and normal tissue. b–d Pathway protein data and analysis, re-analyzed from data first analyzed andreported in our TET1 study.1 Non-tumor samples, N= 8; TNBC/basal-like samples, N = 81; HER2-enriched, N = 53; luminal A, N =208; luminal B, N = 110. Online Resource 2. Alternative confirmation that TET1 isO-GlcNAc modified. a Overexpression of Flag-HA-tagged TET1 catalytic domain in HEK293T and HEK293 cells. Stars indicate TET1-specific bands. b HEK293T cells were fed the azide-labeled per-acetyl galactosamineas a metabolic reporter of O-GlcNAc. Click chemistry was performed with biotinalkyne. HA immunoprecipitation was used to enrich Flag-HA-TET1. Streptavidinblot was used to visualize biotinylated samples. Online Resource 3. Comparison of OGT mRNA levels by qRT-PCRbetween high and low glucose conditions. Media containing high glucosewas added for 24 h following growth in low glucose media. Barsindicate variability across 3 technical replicates for each cell line. Data wascollected for 1 biological replicate per cell line, no significance wasdetermined. OnlineResource 4. SpliceAid diagram of TARDBP binding siteon OGT mRNA. Made using human OGT sequence andhttp://www.introni.it/splicing.html.Online Resource 5a. Gating strategy for analysis of cancer stem-like cell markers. Unstained controls for each condition were used to set the analysis parameters. Front scatter plotand side scatter plotwere use by areaor heightto determine cell morphology. CD44 was analyzed in the PE-Cy7 channel, CD133 was analyzed in the APC channel, and EpCam was analyzedin the Y780 channel to set background. The text below each plot indicates the sample type, then the gating strategy, and the number indicates the total number of events for each run. Cell lines: GFP-vector-expressing MDA-MB-468 cells or MBD2_V2-overexpressing MDA-MB-468 ce [file 12935_2023_2942_MOESM1_ESM.docx]

**Additional file 1**

**Hyperglycemia and O-GlcNAc transferase activity drive a cancer stem cell pathway in triple-negative breast cancer**

Saheed A. Ayodeji,^1^  Bin Bao,^2^ Emily A. Teslow,^2^ Lisa A. Polin,^2^ Greg Dyson,^2^ Aliccia Bollig-Fischer,^2^ Charlie Fehl^1,^*

Affiliations:

^1^ Department of Chemistry, Wayne State University, 5101 Cass Avenue, Detroit, MI, USA

^2^Barbara Ann Karmanos Cancer Institute and Department of Oncology, Wayne State University School of Medicine, Detroit, Michigan, 48201, USA

*charlie.fehl@wayne.edu

**Contents:**

Online Resource 1

Detailed methods for chemical O-GlcNAc labeling

Online Resource 2

Online Resource 3

Online Resource 4

Online Resource 5

Online Resource 6

Full Blot/Gel Images

Primers used for qRT-PCR

Supplemental references

**Online Resource 1**. Expanded TCGA RNA-seq patient sample analysis for CSC pathway genes. **a**) O-GlcNAcase (OGA) levels between subtypes of breast cancer and normal tissue. **b-d**) Pathway protein data and analysis, re-analyzed from data first analyzed and reported in our TET1 study.^1^ Non-tumor samples, N = 8; TNBC/basal-like samples, N = 81; HER2-enriched, N = 53; luminal A, N = 208; luminal B, N = 110.

***Detailed methods for chemoenzymatic O-GlcNAc protein labeling***

*Endogenous O-GlcNAcylated proteins can be labeled in cells using a selective enzymatic addition of azido-galactose analogs followed by copper catalyzed azide-alkyne cycloaddition (CuAAC) “click” reaction to label these glycoproteins. The 4’-OH of the O-GlcNAc sugars react with β – 1,4 galactosyltransferase (GalT). The GalT mutant Y289L accepts the analog UDP-N-azidoacetyl-galactosamine (UDP-GalNAz). Using GalT(Y289L) and UDP-GalNAz, we labeled endogenous O-GlcNAcylated proteins from tissue culture cell lysates as well as from tumor tissues extracted from mice.*


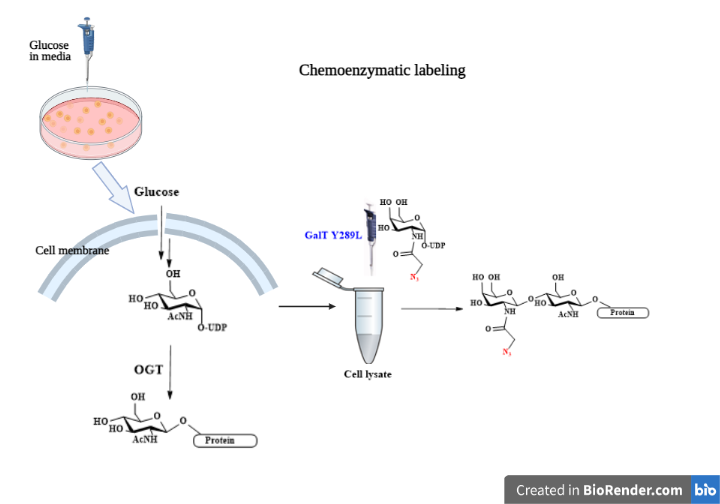


Chemoenzymatic labeling using Click-IT reagent kit

***O-GlcNAc Chemoenzymatic labeling:*** Endogenously modified O-GlcNAc proteins were azide-labeled using the Click-IT^TM^ O-GlcNAc Enzymatic labeling system (ThermoFisher #C33368). Each labeling experiment was started with 200 µg protein lysate (per sample) from cell lines cultured in Low (1.0 g/L) or High (4.5 g/L) Glucose media for 72 hours. The protein lysates in 1.5 mL centrifuge tubes were brought to 200µL using 1% SDS in 20mM HEPES (pH 7.9). Added 600 µL of cold methanol followed by 150 µL of cold chloroform then vortexed for 15 seconds before 400 µL of cold milli-Q water was added to precipitate the proteins. The samples were centrifuged for 5 minutes at 17,000 x g, 4^o^C. The top layer was carefully aspirated off without disturbing the precipitated proteins and the lower organic layer. Added another 450 µL of cold methanol to the samples and carefully tapped the centrifuge tube to resuspend the proteins. These were centrifuged again for 5 minutes at 17, 000 x g, 4^o^C and the supernatant carefully aspirated off. The precipitated proteins were air dried on the laboratory bench for 10 minutes then resuspended in 40 µL of 1% SDS in 20 mM HEPES (pH 7.9). To fully dissolve the proteins, the samples were sonicated in cold water bath at 4^o^C for 15 seconds pulse, three times with a hold of 59 seconds in between pulses. The GalNAz labeling procedure was then performed following the manufacture’s protocol (ThermoFisher), a modified version of the Hsieh-Wilson Lab procedure.^2^


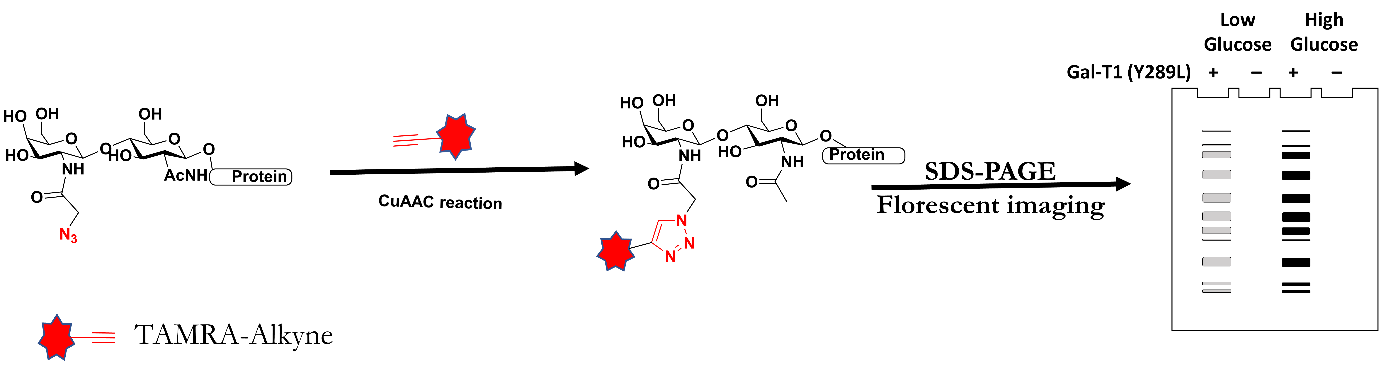


***Copper-catalyzed Azide-Alkyne click reaction:*** The azide labelled lysates were either reacted with TAMRA-alkyne or Biotin alkyne using CuAAC click chemistry. Briefly, azide labelled samples were resuspended in 200uL of 1% NP-40 buffer and sonicated as described above for total dissolution. The click reaction cocktail was made by mixing 5 µL of 2 mM TAMRA-alkyne (or Biotin-alkyne), 2 µL of 10 mM Tris((1-benzyl-4-triazolyl-) methyl) amine (TBTA) and 4 µL of freshly made 50 mM copper (II) sulphate pentahydrate (CuSO_4_.5H_2_O) and gently vortexed for 10 seconds before the addition of 4 µL of freshly prepared 100 mM sodium ascorbate solution in water and vortexed for additional 10 seconds. The 15 µL cocktail was then added to a 1.5 mL centrifuge tube containing the proteins, mounted on a Labquake^TM^ tube rotor, and incubated at lab temperature (approximately 25 ^o^C) for 30 minutes. The proteins were precipitated as described above and washed with additional round of 450 µL cold methanol to get rid of excess alkyne reagent. Tagged proteins were resuspended in 40 µL 1% SDS in 20 mM HEPES (pH 7.9) and sonicated for total dissolution in the buffer.

To visualize total O-GlcNAc proteins, 20 µL of each sample was run on SDS-PAGE (with the addition of appropriate volume of sample buffer and reducing agent) and visualized on fluorescence channel (for TAMRA tagged) or immunoblotted with streptavidin-HRP (for Biotin-labelled samples) on iBright^TM^ FL-1500 imaging system (ThermoFisher).

For protein specific O-GlcNAc detection, biotinylated sample proteins were resuspended in 200 µL NP-40 buffer and enriched with 30 µL of streptavidin coated magnetic beads (NEBS, #S1420S) for 1 hour at 4^o^C with gentle rotation. Flow-through was collected, beads were washed three times with immunoprecipitation buffer, and proteins were eluted with 20 µL of 4X sample buffer, 3 µL of 10X reducing agent and 7 µL of immunoprecipitation buffer for SDS page using magnetic rack. The proteins of interest were transferred and analyzed by western blot.

**Online resource 2**

***Alternative confirmation of TET1 O-GlcNAc modification using metabolic labelling.***

Instead of labeling the endogenous O-GlcNAc modifications of proteins in lysates, azide-modified GlcNAc analogs could be incorporated on O-GlcNAc sites in live cells by incubating the cells with Ac_4_GalNAz, which is converted in cells to UDP-GalNAz and epimerized to UDP-GlcNAz via Galactose epimerase (GALE) as reported.^3^ We synthesized Ac_4_GalNAz following the reported synthesis.^3^ HEK293T cells overexpressing Flag-HA-tagged human TET1 (Addgene #70129) with 200 µM Ac_4_GalNAz for 16 hours. Whole cell protein lysates were collected in cold RIPA buffer and quantified as stated in the main text.


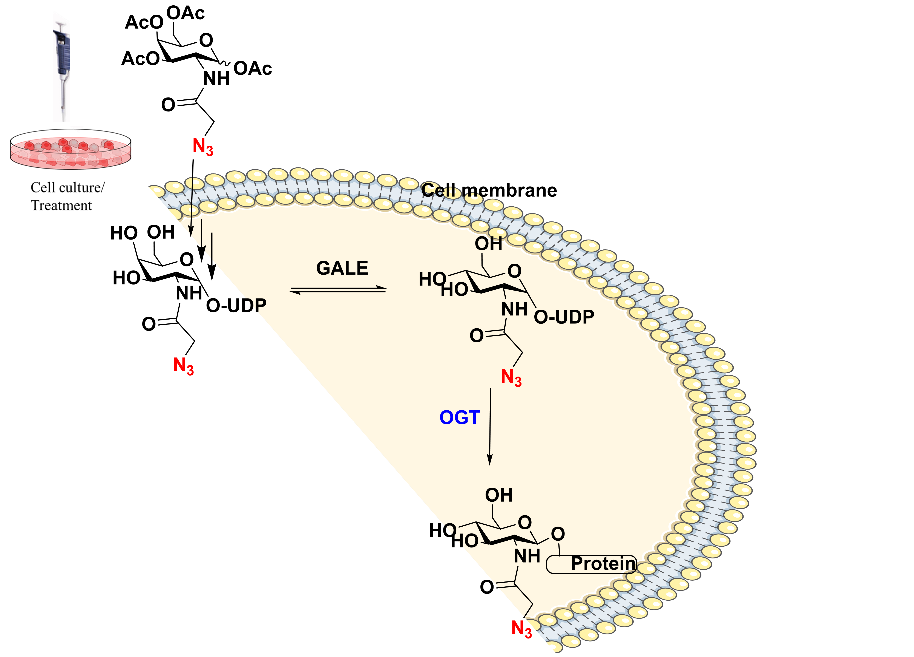


Metabolic labeling approach for O-GlcNAc proteins

SDS-Page (30 µg protein per well) followed by HA or Flag western blotting showed better protein expression in HEK293T cells than HEK293 cells as observed in **Online resource 2A**. The click chemistry was conducated as above with biotin-alkyne substrates. Biotinylated proteins were enriched with streptavidin magnetic beads, followed by SDS-PAGE and immunoblotting with anti-HA antibody revealed TET1 as O-GlcNAc modified in cells (**Online resource 2B**).

**Online Resource 2**. Alternative confirmation that TET1 is O-GlcNAc modified. **A**) Overexpression of Flag-HA-tagged TET1 catalytic domain in HEK293T and HEK293 cells. Stars indicate TET1-specific bands. **B**) HEK293T cells were fed the azide-labeled per-acetyl galactosamine (Ac_4_-GalNAz) as a metabolic reporter of O-GlcNAc. Click chemistry was performed with biotin alkyne. HA immunoprecipitation was used to enrich Flag-HA-TET1. Streptavidin blot was used to visualize biotinylated samples (O-GlcNAc proteins).

**Online Resource 3**. Comparison of OGT mRNA levels by qRT-PCR between high and low glucose conditions. Media containing high glucose (4.5 g/L) was added for 24 h following growth in low glucose media (1.0 g/L). Bars indicate variability across 3 technical replicates for each cell line. Data was collected for 1 biological replicate per cell line, no significance was determined.

**Online Resource 4:**

The SpliceAid tool (http://www.introni.it/splicing.html) is an experimentally-validated database of RNA-protein interactions.^4^ We input the OGT mRNA into SpliceAid and generated the map in **Online Resource 3**, which revealed a TARDBP binding site. We hypothesize that this TARDBP-OGT(mRNA) interaction regulates OGT levels in cells based on our knockdown studies in the main text.


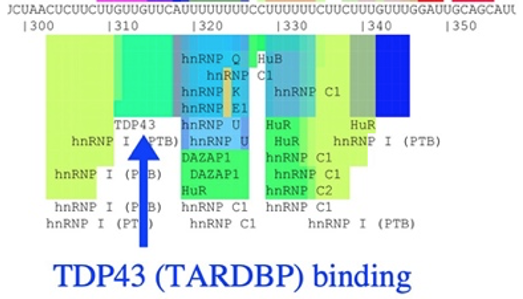


**Online Resource 4**. SpliceAid diagram of TARDBP binding site on OGT mRNA. Made using human OGT sequence and http://www.introni.it/splicing.html.

**Online Resource 5:**

Fluorescent cell analysis was performed to quantify stem cell markers following OGT inhibition. As a rescue construct, the protein MBD2_v2 was stably overexpressed (“V2”) compared to the control cells, which stably overexpressed GFP (“GFP”). We analyzed CD44, EpCam, and CD133 as known markers for cancer stem-like cells in TNBC.^5,6^ For each condition, the left plot shows how cells first gated for front scatter and CD44 expression, then the second plot shows EpCam and CD133 analysis. The triple-marker positive population is listed in the top-right quadrant of the EpCam/CD133 plot (**Online Resource 5**). We noted that Control (no treatment) and OSMI-4 treated cells (10 uM, 48 h treatment) showed slight morphology differences via front and side-scatter plots, which were used to set the appropriate gates in the following experiments.

**Online Resource 5a**. Gating strategy for analysis of cancer stem-like cell markers. Unstained controls for each condition were used to set the analysis parameters. Front scatter plot (FSC) and side scatter plot (SSC) were use by area (A) or height (H) to determine cell morphology. CD44 was analyzed in the PE-Cy7 channel, CD133 was analyzed in the APC channel, and EpCam was analyzed in the Y780 channel to set background. The text below each plot indicates the sample type, then the gating strategy (i.e. for single cells as determined by FSC/SSC plots), and the number indicates the total number of events for each run. Cell lines: GFP-vector-expressing MDA-MB-468 cells or MBD2_V2-overexpressing MDA-MB-468 cell lines.

**Online Resource 5b**. Analysis of cancer stem-like cell markers CD44, CD133, and EpCam in bulk TNBC cells. Cell lines: stable GFP-vector-expressing MDA-MB-468 cells were treated with DMSO control. Cells were first gated by CD44, then analyzed for CD133 and EpCam staining. All three markers represent cancer stem-like cells. Results presented for three biological replicates.

**Online Resource 5c**. Analysis of cancer stem-like cell markers CD44, CD133, and EpCam in bulk TNBC cells. Cell lines: stable GFP-vector-expressing MDA-MB-468 cells were treated with OGT inhibitor (OSMI-4, 10 uM, 48 h). Cells were first gated by CD44, then analyzed for CD133 and EpCam staining. All three markers represent cancer stem-like cells. Results presented for three biological replicates.

**Online Resource 5d**. Analysis of cancer stem-like cell markers CD44, CD133, and EpCam in bulk TNBC cells. Cell lines: stable MBD2v2-overexpressing MDA-MB-468 cells were treated with DMSO control. Cells were first gated by CD44, then analyzed for CD133 and EpCam staining. All three markers represent cancer stem-like cells. Results presented for three biological replicates.

**Online Resource 5e**. Analysis of cancer stem-like cell markers CD44, CD133, and EpCam in bulk TNBC cells. Cell lines: stable MBD2v2-overexpressing MDA-MB-468 cells were treated with OGT inhibitor (OSMI-4, 10 uM, 48 h). Cells were first gated by CD44, then analyzed for CD133 and EpCam staining. All three markers represent cancer stem-like cells. Results presented for three biological replicates.

**
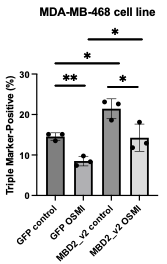
**

**Online Resource 5f**. Analysis of cancer stem-like cell markers CD44, CD133, and EpCam in bulk TNBC cells. Bars indicate the standard error of the mean of three biological replicates. Statistical analysis: * indicates P ≤ 0.05, ** indicates P ≤ 0.01.

**Online Resource 6.** Weight of mice on high fat diet (60% fat) relative to control diet (10% fat). The difference was significant at 5 weeks, P < 0.001. 12 mice per group.

**Full Blot/Gel Images**

**Figure 2 uncropped images**


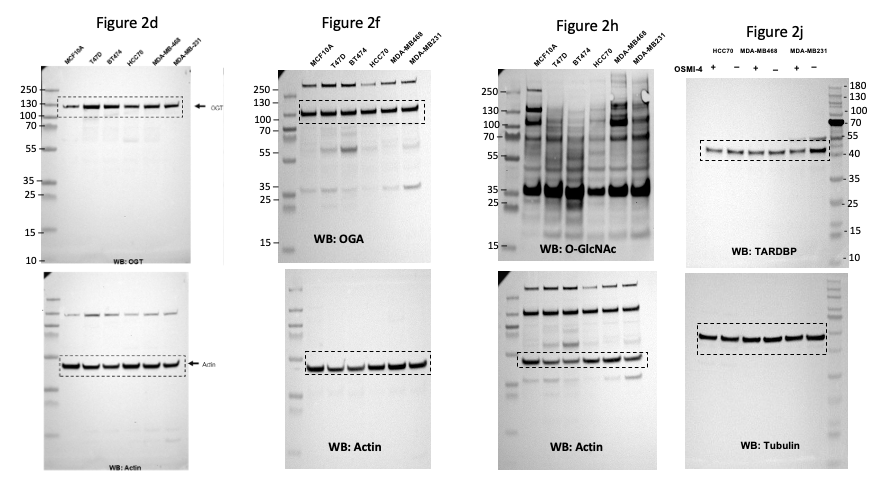


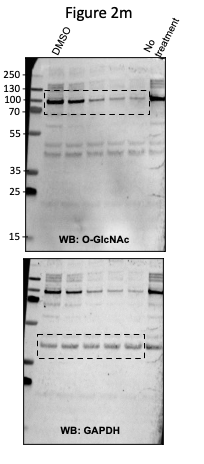


**Figure 3 uncropped images**


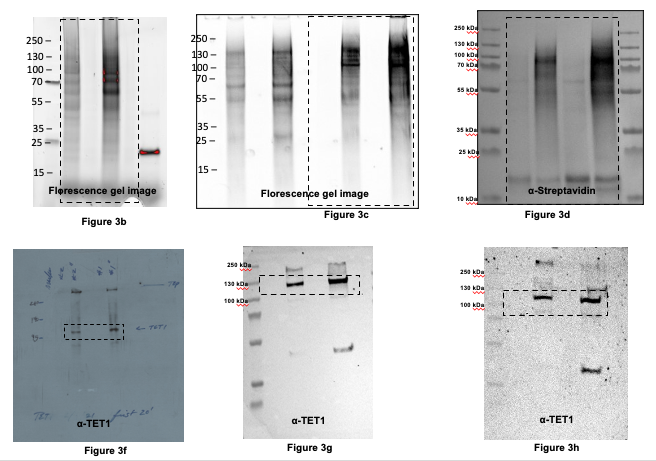


Chemoenzymatic labeling, O-GlcNAc protein Visualization and TET1 Immunoblotting from HCC70, MDA-MB-468 and MDA-MB-231 cell protein lysates (left to right).

**Figure 5, uncropped images**


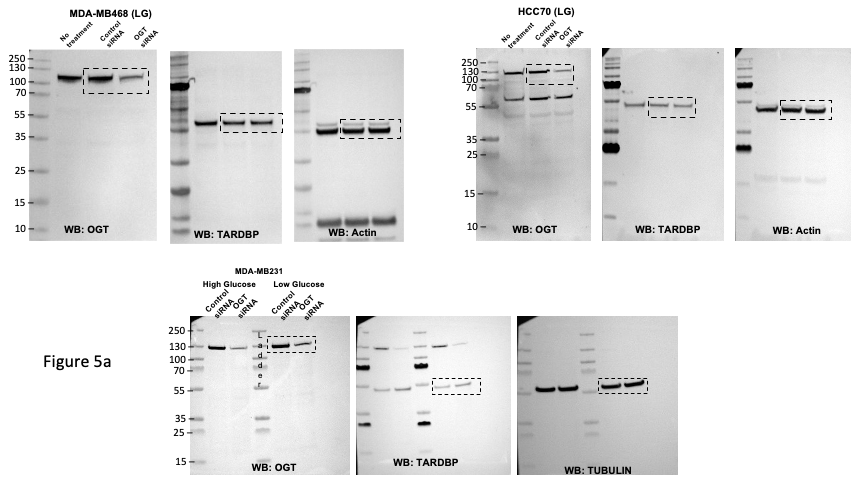


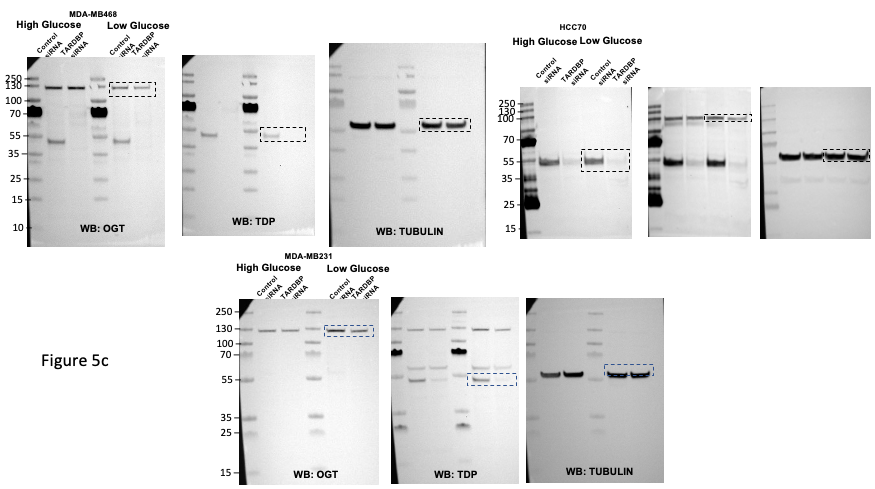


**Figure 7e uncropped image**

**
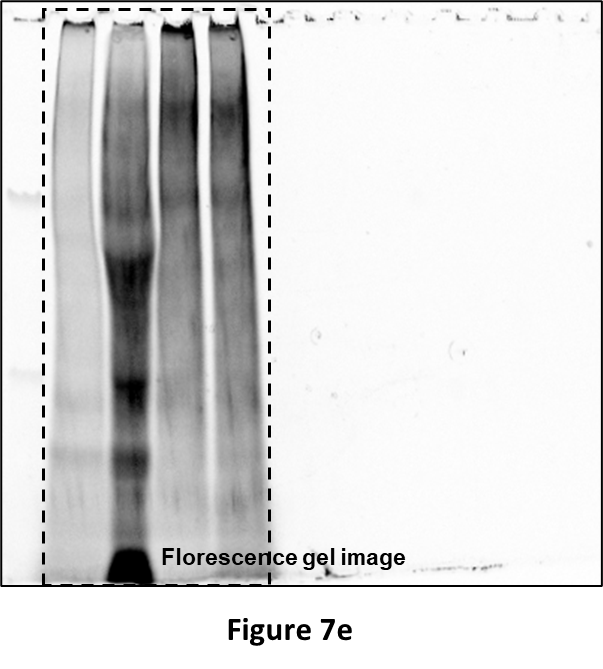
**

Chemoenzymatic labeling in mouse tissue. Left to right: lean/cytosolic; DIO/cytosolic; lean/nuclear; DIO/nuclear proteins.

**qRT-PCR Primers**

*The following primers were used for qRT-PCR of OGT mRNA expressions based on Walker et. al.^7^*

OGT sense: 5’- ACTGTGTTCGCAGTGACCTG-3’

OGT antisense: 5’-CAAATTTCCCCTTGTGCATT-3’

*The primers for TET1, OGA, TARDBP, SRSF2, MBD2_V2 and NANOG were purchased from Integrated DNA Technologies Inc. (Coralville, IA) or Fisher Scientific as reported in our previous studies.^1,6,8^ PUM1 and Actin were selected as loading controls based on breast cancer normalization data.^9^*

Beta-Actin forward: CCCAGCACAATGAAGATCAA

Beta-Actin reverse: ACATCTGCTGGAAGGTGGAC

PUM1 forward: AGTGGGGGACTAGGCGTTAG

PUM1 reverse: GTTTTCATCACTGTCTGCATCC

OGA forward: TCTTGTACACCGATGCCTCA

OGA reverse: AGAACCCTGGGCCTTTAGAG

TET1 forward: CGCTACGAAGCACCTCTCTTA

TET1 reverse: CTTGCATTGGAACCGAATCATTT

TARDBP forward: AATGAGGAACAGAGGGAAAC

TARDBP reverse: GCTGAACCTGAAGACTGAATA

SRSF2 forward: CCCGATGTGGAGGGTATGAC

SRSF2 reverse: GAGACTTCGAGCGGCTGTAG

PrimerBankID: 306482644c1

MBD2v2 – TaqMan assay, Fisher Scientific # Hs00210557

NANOG forward: CCCCAGCCTTTACTCTTCCTA

NANOG reverse: CCAGGTTGAATTGTTCCAGGTC

PrimerBankID: 153945815c3

**References**

1. Bao, B.; Teslow, E. A.; Mitrea, C.; Boerner, J. L.; Dyson, G.; Bollig-Fischer, A., Role of TET1 and 5hmC in an Obesity-Linked Pathway Driving Cancer Stem Cells in Triple-Negative Breast Cancer. *Molecular Cancer Research* **2020,** *18* (12), 1803-1814.

2. Thompson, J. W.; Griffin, M. E.; Hsieh-Wilson, L. C., Chapter Four - Methods for the Detection, Study, and Dynamic Profiling of O-GlcNAc Glycosylation. In *Methods in Enzymology*, Imperiali, B., Ed. Academic Press: 2018; Vol. 598, pp 101-135.

3. Boyce, M.; Carrico, I. S.; Ganguli, A. S.; Yu, S.-H.; Hangauer, M. J.; Hubbard, S. C.; Kohler, J. J.; Bertozzi, C. R., Metabolic cross-talk allows labeling of O-linked β-N-acetylglucosamine-modified proteins via the N-acetylgalactosamine salvage pathway. *Proceedings of the National Academy of Sciences* **2011,** *108* (8), 3141-3146.

4. Piva, F.; Giulietti, M.; Nocchi, L.; Principato, G., SpliceAid: a database of experimental RNA target motifs bound by splicing proteins in humans. *Bioinformatics* **2009,** *25* (9), 1211-3.

5. Ricardo, S.; Vieira, A. F.; Gerhard, R.; Leitão, D.; Pinto, R.; Cameselle-Teijeiro, J. F.; Milanezi, F.; Schmitt, F.; Paredes, J., Breast cancer stem cell markers CD44, CD24 and ALDH1: expression distribution within intrinsic molecular subtype. *J Clin Pathol* **2011,** *64* (11), 937-46.

6. Bao, B.; Mitrea, C.; Wijesinghe, P.; Marchetti, L.; Girsch, E.; Farr, R. L.; Boerner, J. L.; Mohammad, R.; Dyson, G.; Terlecky, S. R.; Bollig-Fischer, A., Treating triple negative breast cancer cells with erlotinib plus a select antioxidant overcomes drug resistance by targeting cancer cell heterogeneity. *Sci Rep* **2017,** *7*, 44125.

7. Tan, Z.-W.; Fei, G.; Paulo, J. A.; Bellaousov, S.; Martin, S. E. S.; Duveau, D. Y.; Thomas, C. J.; Gygi, S. P.; Boutz, P. L.; Walker, S., O-GlcNAc regulates gene expression by controlling detained intron splicing. *Nucleic acids research* **2020,** *48* (10), 5656-5669.

8. Teslow, E. A.; Mitrea, C.; Bao, B.; Mohammad, R. M.; Polin, L. A.; Dyson, G.; Purrington, K. S.; Bollig-Fischer, A., Obesity-induced MBD2_v2 expression promotes tumor-initiating triple-negative breast cancer stem cells. *Mol Oncol* **2019,** *13* (4), 894-908.

9. Kılıç, Y.; Çelebiler, A.; Sakızlı, M., Selecting housekeeping genes as references for the normalization of quantitative PCR data in breast cancer. *Clinical & translational oncology : official publication of the Federation of Spanish Oncology Societies and of the National Cancer Institute of Mexico* **2014,** *16* (2), 184-90.
